# Phase Equilibria and Crystal Chemistry in Portions of the System SrO-CaO-Bi_2_O_3_-CuO, Part II—The System SrO-Bi_2_O_3_-CuO

**DOI:** 10.6028/jres.095.029

**Published:** 1990

**Authors:** R. S. Roth, C. J. Rawn, B. P. Burton, F. Beech

**Affiliations:** National Institute of Standards and Technology, Gaithersburg, MD 20899

**Keywords:** crystal chemistry, phase equilibria, single crystal diffraction, SrO-CaO-Bi_2_O_3_-CuO, superconductivity, *x*-ray powder diffraction

## Abstract

New data are presented on the phase equilibria and crystal chemistry of the binary systems Sr0-Bi_2_0_3_ and SrO-CuO and the ternary system SrO-Bi_2_O_3_-CuO. Symmetry data and unit cell dimensions based on single crystal and powder x-ray diffraction measurements are reported for all the binary SrO-Bi_2_O_3_ phases, including a new phase identified as Sr_6_Bi_2_O_9_. The ternary system contains at least four ternary phases which can be formed in air at ~900 °C. These are identified as Sr_2_Bi_2_CuO_6_, Sr_8_Bi_4_Cu_5_O_19+_*_x_*, Sr_3_Bi_2_Cu_2_O_8_ and a solid solution (the Raveau phase) which, for equilibrium conditions at ~900 °C, corresponds approximately to the formula Sr_1.8−_*_x_*Bi_2.2+_*_x_*Cu_1±_*_x_*_/2_O*_z_*.(0.0⩽*x*⩽~0.15). Superconductivity in this phase apparently occurs only in compositions that correspond to negative values of *x*. Compositions that lie outside the equilibrium Raveau-phase field often form nearly homogeneous Raveau-phase products. Typically this occurs after relatively brief heat treatments, or in crystallization of a quenched melt.

## 1. Introduction

The discovery of high transition temperature (*T*_c_) superconductivity in cuprates by Bednorz and Müller [1] and its confirmation by Takagi et al. [2] as being due to the phase La_2−_*_x_*Ba*_x_*CuO_4_ led to a world-wide search for other compounds with higher *T*_c_. These researches first produced La_2−_*_x_*Sr*_x_*CuO4 [3] and quickly led to the discovery of a mixed phase composition in the system BaO-Y_2_O_3_-CUO with a *T_c_*~90 K [4], well above Uquid nitrogen temperature (73 K). Identification of the superconducting phase as Ba_2_YCu_3_O_6+_*_x_* [5] has resulted in hundreds of published reports on the properties of this phase. Our own phase equilibria studies of the system BaO-Y_2_O_3_-CuO [6, 7] have shown that CO_2_ is an important constituent of bulk ceramics that are prepared in air.

Phases with still higher *T*_c_ were found in the systems SrO-CaO-Bi_2_Os-CuO and BaO-CaO-Tl_2_O_3_-CuO [8, 9]. These phases belong mostly to a homologous series A_2_Ca*_n_*_−1_B_2_Cu*_n_*O_2_*_n_*_+4_ (A= Sr, Ba; B= Bi, Tl) although another series A_2_Ca*_n_*_−1_BCu*_n_*O_2_*_n_*_+3_ (A = Ba, B= Tl) can also lead to superconducting phases [10]. Still other compounds have been discovered with high *T*_c_, i.e., Pb_2_Sr_2_YCu_3_O_8+δ_ [11], Ba_1−_*_x_*K*_x_*BiO_3_ [12] (with no Cu ions!) and Nd_2−_*_x_*Ce*_x_*CuO_4_ [13]. The Tl^+3^ containing phases with the largest values of *n*, so far have the highest confirmed *T*_c_, up to ~125 K [9]. However, the phases in the Tl^+3^ system are difficult to prepare as bulk single phase samples, and the relevant phase equilibria have not been determined, owing to the extreme volatility of Tl and the poisonous nature of Tl vapors. In the Bi^+3^ containing systems the phase with *n*=2 and *T*_c_~80 K is easily prepared. However, its exact single-phase region is not well known and a structure determination has not been completed because of very strong incommensurate diffraction that is apparently due to a modulation of the Bi positions. Higher *n* (and higher *T*_c_ phases have not been prepared as single phase bulk specimens (without PbO). Thus, we undertook a comprehensive study of the phase equilibria and crystal chemistry of the entire four component system SrO-CaO-Bi_2_O_3_-CuO. It is hoped that a complete understanding of the crystal chemistry and thermodynamics of the many phases formed will lead to a better understanding of the processing parameters for the preparation of bulk ceramics with reproducible and useful properties.

A prerequisite to understanding the phase equilibria of the four-component system is adequate definition of the phase relations in the bounding binary and ternary systems. The ternary system SrO-CaO-CuO was the first to be investigated and the results were pubhshed separately [14]. The solubilities of CaO in the solid solutions that are based on SrO:CuO phases were determined, and a ternary phase Ca_1−_*_x_*, Sr*_x_*CuO_2_ (*x* =0.14–0.16) was discovered. The structure of this ternary phase was refined by Siegrist et al. [15]. The present paper discusses the experimental determination of the phase relations and crystal chemistry of the ternary system SrO-Bi_2_0_3_-CuO as well as its boundary binary systems. A portion of the binary SrO-CuO system was previously published [16], and the structure of the compound “Sr_14_Cu_24_O_41_” was determined [17]. Because of the relative importance of the phase Sr_2_Bi_2_CuO_6_, a separate paper was prepared concerning the composition, unit cell dimensions and symmetry of this phase [18]. The experimental details, phase relations and crystal chemistry of the binary CaO-Bi_2_O_3_ and the two remaining ternary systems CaO-Bi_2_O_3_-CuO and SrO-CaO-Bi_2_O_3_ are reported in separate publications [19,20].

In the following discussion of phase equilibria and crystal chemistry, the oxides under consideration will always be given in the order of decreasing ionic radius, largest first, e.g., $\mathrm{SrO}:\frac{1}{2}{\mathrm{Bi}}_{2}O_{3}:\mathrm{CuO}$. The notation $\frac{1}{2}{\mathrm{Bi}}_{2}O_{3}$ is used so as to keep the metal ratios the same as the oxide ratios. The standard cement/ceramic notation is used for short hand with $S=\mathrm{SrO},B=\frac{1}{2}{\mathrm{Bi}}_{2}O_{3}$ and C=CuO. Thus compositions may be listed simply by numerical ratio, e.g., the formula Sr_2_Bi_2_CuO_6_ can be written as S_2_B_2_C or simply 2:2:1.

## 2. Experimental Procedures

In general, about 3.5 g specimens of various compositions in binary and ternary combinations were prepared from SrCO_3_, Bi_2_O_3_, and CuO. Neutron activation analyses of the starting materials indicated that the following impurities (in *μ*g/g) were present: in CuO−3.9Cr, 2.8Ba, 28Fe, 410Zn, 0.09Co, 1.9Ag, 0.03Eu, 14Sb; in Bi_2_O_3_−2.1Cr, 0.0002Sc, 26Fe, 21Zn, 0.6Co, 0.5Ag, 0.0008Eu, 0.2Sb; in SrCO_3_−320Ba, 0.001Sc, 6.3Fe, 3.7Zn, 0.1 Co, 0.002Eu. The constituent chemicals were weighed on an analytical balance to the nearest 0.0001 g and mixed either dry or with acetone in an agate mortar and pestle. The weighed specimen was pressed into a loose pellet in a stainless steel die and fired on an MgO single crystal plate, or on Au foil, or on a small sacrificial pellet of its own composition. The pellets were then calcined several times at various temperatures from ~ 600 °C to 850 °C, with grinding and repelletizing between each heat treatment. Duration of each heat treatment was generally about 16–20 h. For the final examination a small portion of the calcined specimen was refired at the desired temperature (1–8 times), generally overnight, either as a small pellet or in a small 3 mm diameter Au tube, either sealed or unsealed. Too many heat treatments in the Au tube generally resulted in noticeable loss of Cu to the Au vessel.

When phase relations involving partial melting were investigated, specimens were contained in 3 mm diameter Au, Pt or Ag/Pd tubes and heated in a vertical quench furnace. This furnace was heated by six MoSi_2_ hairpin heating elements with vertical 4-in diameter ZrO_2_ and 1-in diameter Al_2_O_3_ tubes acting as insulators. The temperature was measured separately from the controller at a point within approximately 1 cm of the specimen by a Pt/90Pt10Rh thermocouple, calibrated against the melting pomts of NaCl (800.5 °C) and Au (1063 °C). After the appropriate heat treatment the specimen was quenched by dropping it into a Ni crucible, which was cooled by He flowing through a copper tube immersed in liquid N_2_.

In order to approach equilibrium phase boundaries by different synthesis routes, many specimens were prepared from pre-made compounds or two-phase mixtures as well as from end members. These were weighed, mixed and ground in the same way as for the previously described specimens. Also, some specimens were: 1) annealed at some temperature (*T*_1_) and analyzed by x-ray powder diffraction; 2) annealed at a higher or lower temperature (*T*_2_) where a different assemblage of phases was observed; and 3) returned to *T*_1_ to demonstrate reversal of the reaction(s) between *T*_1_ and *T*_2_. All experimental details are given in tables 1a and 1b. Phase identification was made by x-ray powder diffraction using a high angle diffractometer with the specimen packed into a 5 or 10 mil deep cavity in a glass slide. The diffractometer, equipped with a theta compensating slit and a graphite diffracted beam monochromator, was run at $\frac{1}{4}{}^{\circ}2\theta$/min with CuKα radiation at 40 KV and 30 MA. The radiation was detected by a scintillation counter and solid state amplifier and recorded on a chart with 1°2*θ* = 1 in. For purposes of illustration and publication, the diffraction patterns of selected specimens were collected on a computer-controlled, step scanning goniometer and the results plotted in the form presented.

Equilibrium in this system has proven to be so difficult to obtain that a few specimens were prepared by utilizing an organic precursor route to obtain more intimate mixtures at low temperatures. It is relatively simple to make mixtures of SrO (with or without CaO) and CuO by utilizing acetate solutions or acrylic acid, but Bi_2_O_3_ is not soluble in these solutions. The carbonates of all three (or four) oxides were therefore dissolved in lactic acid and dried by slow heating in a container with a large surface-to-volume ratio. This procedure yields an essentially single phase amorphous precursor for all compositions that contain less than about 66.7 mole percent Bi_2_O_3_. At higher bismuth contents, pure Hi metal was formed by carbothermic reduction under even the lowest temperature drying procedures in air.

## 3. Experimental Results and Discussion

Most of the experiments performed on the binary and ternary mixtures of SrO:Bi_2_O_3_:CuO are reported in table 1a. Additional experiments specifically designed in an attempt to obtain crystals large enough for x-ray single crystal study are detailed in table 1b. Crystallographic data for various phases are reported in table 2.

### 3.1 The System Bi_2_O_3_-CuO

A phase diagram for this system was already published [21], and was redrawn as figure 6392 in Phase Diagrams for Ceramists (PDFC) [22]. It apparently contains only one compound, Bi_2_CuO_4_ (B_2_C), which is tetragonal, space group P4/ncc, *a* = 8.510, *c* = 5.814 Å [23]. The x-ray powder diffraction data for Bi_2_CuO_4_ were also reported in [23]. The very limited number of experiments performed during the course of this work, as shown in table 1, confirms that this is the only compound formed in the system. No attempt was made to reinvestigate the melting relations of this system because it does not have any great effect on the phase equilibria of the ternary system with SrO.

### 3.2 The System SrO-CuO

**Figure 1:**
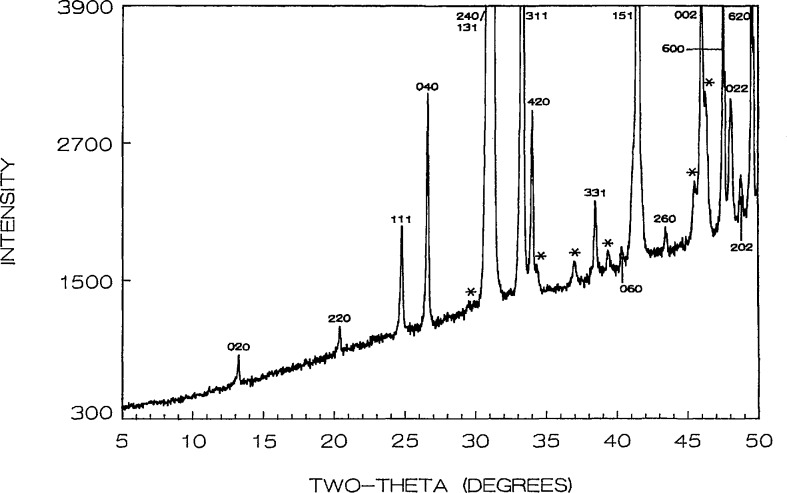
X-ray powder diffraction pattern of Sr_14_Cu_24_O_41_ (cooled from 925 °C). *Superstructure peaks.

Phase equilibria in the high CuO portion of the system were shown in [16], where the new compound “Sr_14_Cu_24_O_41_” (S_14_C_24_) was proven to exist along with the previously reported SrCuO_2_ [24] and Sr_2_CuO_3_ [25]. Refined unit cell dimensions and standard x-ray powder diffraction data for the last two phases were recently reported: SrCuO_2_ (SC) [26] is orthorhombic (Cmcm) with *a* =3.5730(2), *b*=16.3313(8), *c* = 3.9136(2) Å; Sr_2_CuO_3_ (JCPDS 34-283) is also orthorhombic (Immm) *a* =3.4957, *b* = 12.684, *c* = 3.9064 A. The unit cell dimensions of Sr_14_Cu_24_O_41_ (S_14_C_24_) [16, 17] indicate that it is face centered orthorhombic with *a* = 11.483(1), *b* = 13.399(1) and *c* = 3.9356(3) Å; there are also some superstructure peaks in the pattern which may possibly be indexed on an incommensurate cell that has a *c*-axis which is about 7 times that of the subcell. The partially indexed x-ray powder diffraction data is given in table 3 and the pattern is illustrated in figure 1.

**Figure 2:**
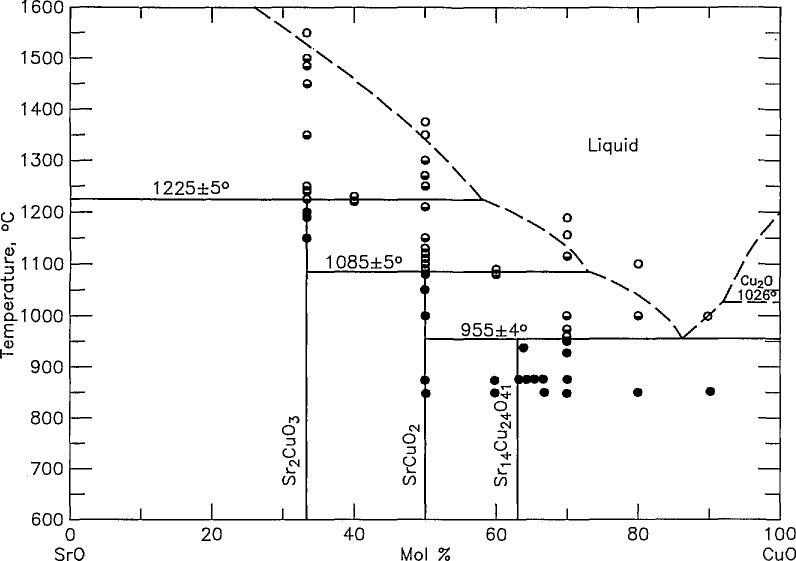
Phase diagram for the system SrO-CuO ●-not melted, ◒-partially melted, ○-completely melted.

Determinations of the melting relations in the high-SrO portion of the system were complicated by charge-capsule reactions (table 1). Specimens of SrCuO_2_ and Sr_2_CuO_2_ (SC and S_2_C) were calcined to single phase and then small portions reheated in 3-mm diameter unsealed Pt tubes; Au capsules could not be used because the melting points of interest were higher than that of Au (1063 °C). Even though these experiments had a maximum duration of no more than 10 min at high-temperature, some CuO always alloyed with the Pt even at temperatures well below melting. Partial melting was assumed to have occurred when the x-ray powder diffraction pattern of a quenched specimen indicated an abrupt change in the phase fraction of a second phase. Both SrCuO_2_ and Sr_2_CuO_3_ melt incongruently: SrCuO_2_ melts to liquid plus Sr_2_CuO_3_ at ~ 1085 °C, and Sr_2_CuO_3_ melts to liquid plus SrO, at ~ 1225 °C. The phase equilibria diagram constructed from the data in table 1 and the previously reported experiments [16] is shown in figure 2.

### 3.3 The System SrO-Bi_2_O_3_

**Figure 3:**
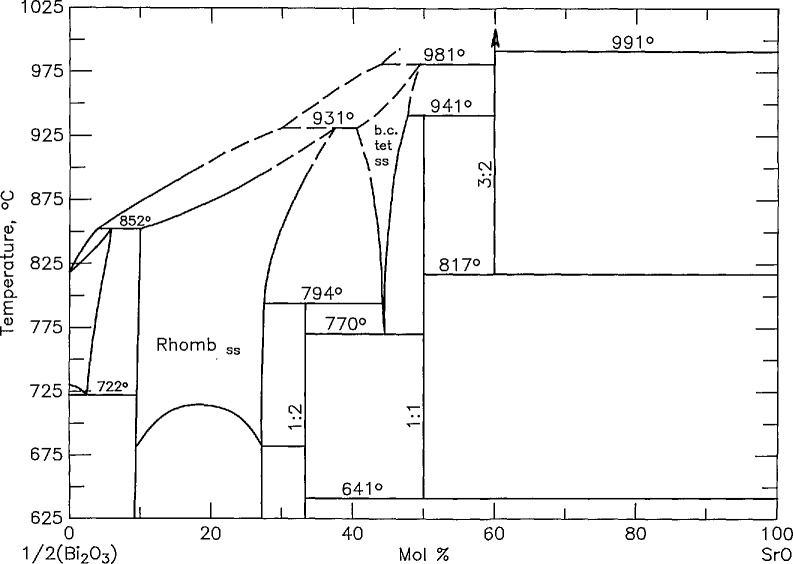
Phase diagram for the system $\mathrm{SrO}-\frac{1}{2}{\mathrm{Bi}}_{2}O_{3}$ modified from that published in [27].

The phase equilibria diagram for the system SrO-Bi_2_O_3_ was reported in [27] and redrawn as figure 6428 m PDFC [22] and figure 3 (where the scale is changed to $\frac{1}{2}{\mathrm{Bi}}_{2}O_{3}:\mathrm{CuO}$ instead of the original Bi_2_O_3_:CuO, to be consistent with the other phase diagrams in this report). Considerable effort was made to study the phase relations of this binary. Complete experimental results are published in [28], and the results are shown in figures 4a and 4b (compare with fig. 3). The major differences between our new diagram and the one presented in [27] are: 1) the occurrence of a new compound which is estimated to have the stoichiometry Sr_6_Bi_2_O_9_ (fig. 4a); 2) the presence of a high temperature polymorph of SrBi_2_O_4_ (fig. 4b) which becomes stable between 800 and 825 °C and melts incongruently at 940±5 °C; and 3) the determination of melting relations in the region of 20–50 mol percent SrO.

#### 3.3.1 Rhombohedral Solid Solution (Sillen Phase-Rhomb)

The rhombohedral solid solution was first reported by Sillen [29] and it was later shown by Levin and Roth [30] that the solidus temperature is increased when SrO is added to face-centered-cubic (fee) Bi_2_O_3_, or when it is added to the rhombohedral solid solution phase. Melting relations in the SrO-rich region of the Sillen phase field were previously [27] represented schematically (by dashed lines) as a melting loop, but the experiments reported in [28] indicate a congruent melting point between 25–30 mol% SrO and 950–960 °C. Guillermo et al. [27] reported that a phase transition occurred from one rhombohedral phase to another, but as this has not been confirmed by quench data, such possible polymorphism is ignored in the present work. X-ray diffraction data for this phase are well established [27] and will not be summarized here.

#### 3.3.2 SrBi_2_O_4_ (SB_2_)

**Figure 5:**
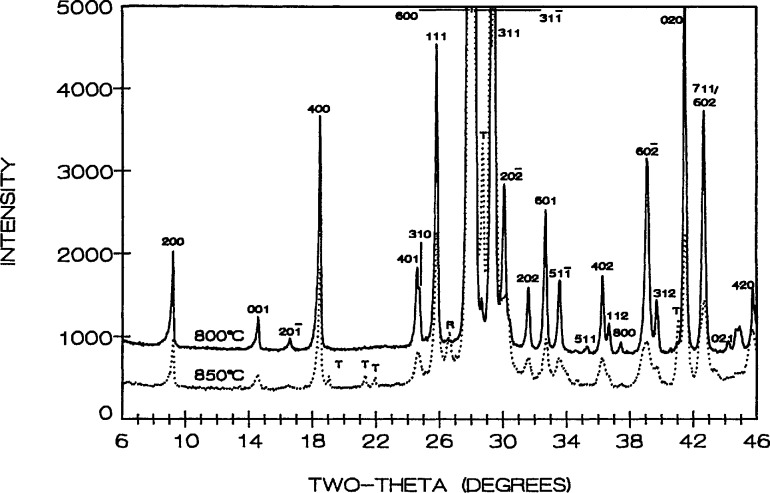
X-ray powder diffraction patterns for low-temperature (cooled from 800 °C) solid line and high-temperature SrBi_2_O_4_ (cooled from 850 °C) dotted line. T=tetragonal phase, R=rhombohedral phase.

SrBi_2_O_4_ appears to have both high- and low-temperature polymorphs with a transition point at about 825 °C. The high-temperatures form melts incongruently to liquid plus the tetragonal solid solution (next section) between 940 and 945 °C. In the high to low-temperature transition, sharp x-ray diffraction peaks in a powder pattern of the low-temperature phase become broad and diffuse when specimens are quenched from about 825–940 °C. Also, a few maxima (e.g., 202) that are present in patterns from the low-temperature phase have drastically reduced intensities in patterns from samples that were quenched from above 825 °C. The indexed x-ray powder diffraction data for low-SrBi_2_O_4_ are listed in table 4. The patterns for both low-temperature and high-temperature SrBi_2_O_4_ are shown in figure 5. The observed broadening of diffraction maxima in the pattern from the quenched sample suggests that the high-temperature polymorph, perhaps orthorhombic, was not successfully quenched. The presence of broad rather than sharp peaks suggests a small domain size in samples quenched from above 825 °C.

**Figure 6:**
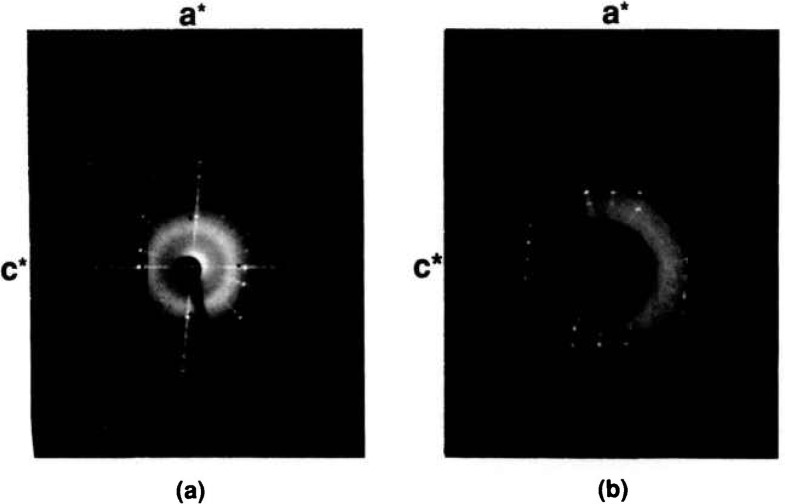
X-ray precession photographs for SrBi_2_O_4_ (a) *h0l*, (b) *hIl.*

Very small single crystals of low-temperature SrBi_2_O_4_ were prepared (table 1b) by heating a prereacted powder sample of SrBi_2_O_4_ plus a 1:1 NaCl:KCl fiux (80/20 flux/charge ratio) in a sealed Pt tube. The specimen was heated to 740 °C and cooled to 570 °C at 6 °/h. After the flux was dissolved with H_2_O, a very thin flat platelet was picked and single crystal x-ray precession photographs were taken (fig. 6) of it. The precession data indicate that the phase is C-centered monoclinic, probably C2/m, and unit cell dimensions refined from x-ray powder diffraction data are *a*=19.301(2), *b*=4.3563(5), *c*=6.1049(7) Å, *β*=94.85(1)°. Larger crystals were obtained from both 80:20 and 50:50 flux/charge ratios by cooling from 800 °C to 645 °C at 1°/h.

#### 3.3.3 The Tetragonal Solid Solution Near SrBi_1.22_O_2,83_ (Tet)

This phase was previously reported [27] with space group I4/m, *a* = 13.239(2), *c*=4.257(1) Å. Experiments during the course of this study agree reasonably well with those previously reported, except for the region near the solidus where we find the single phase region extends to compositions with at least 50 mol percent SrO. The x-ray powder diffraction data was previously reported [27]. Very large single crystals were obtained by cooling the Sr_2_Bi_2_O_5_ composition from above the melting point to ~950 °C.

#### 3.3.4 Sr_2_Bi_2_O_5_(S_2_B_2_)

**Figure 7:**
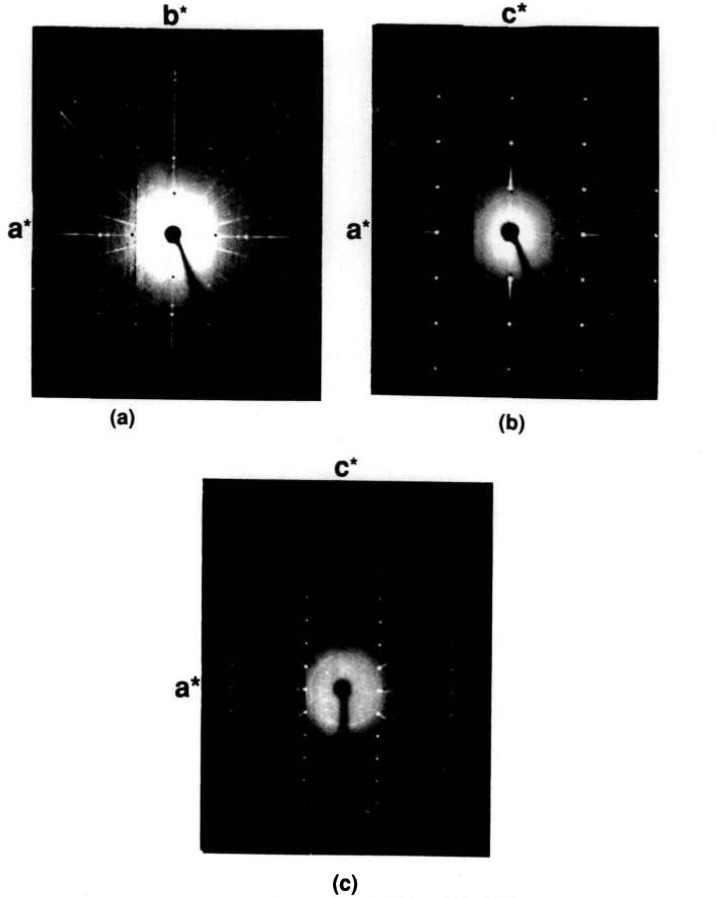
X-ray precession photographs of Sr_2_Bi_2_O_5_ (a) *hk0*, (b) *h0l* and (c) *hIl.*

**Figure 8:**
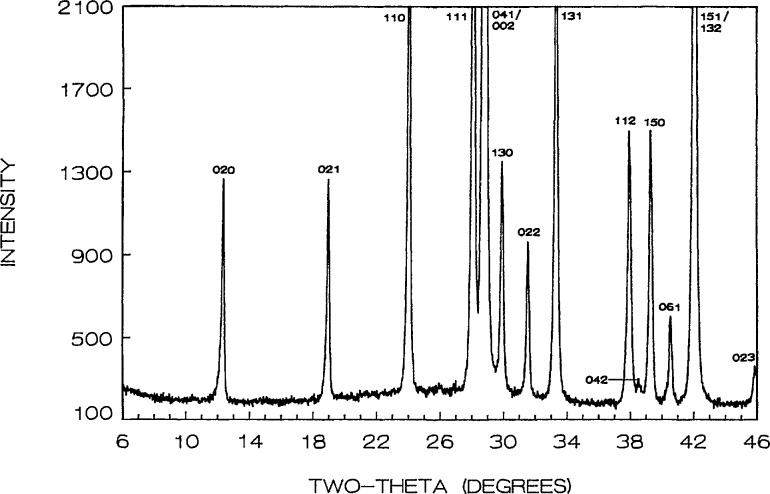
X-ray powder diffraction pattern of Sr_2_Bi_2_O_5_ (cooled from 900 °C).

The compound Sr_2_Bi_2_O_5_ was reported [27] to be orthorhombic, space group Pcmm with *a* = 14.293(2), *b*=7.651(2) and *c* =6.172(1) Å. Although precession photographs collected from very small crystals in the present study show evidence of only $\frac{1}{2}$ the *b* axis reported in [27] (see fig. 7), much larger crystals showed a very weak superstructure and a doubled *b*-axis. The subcell space group is apparently Cmcm and in this orientation *a* =3.8262(2), *b* = 14.307(1), *c* = 6.1713(4) Å as obtained from a least-squares refinement of the powder data. The indexed powder data are given in table 5 and illustrated in figure 8. Apparently the superstructure destroys the subcell symmetry of the C-centering, showing such peaks as (1/2, 16, 0) and (1 1/2, 0, 1) resulting in a space group symmetry consistant with Pbnm. Very large single crystals were obtained by cooling the Sr_2_Bi_2_O_5_ composition from above the melting point to ~900°C, and annealing large fragments at 850 °C—258 h.

#### 3.3.5 Sr_3_Bi_2_O_6_(S_3_B_2_)

**Figure 9:**
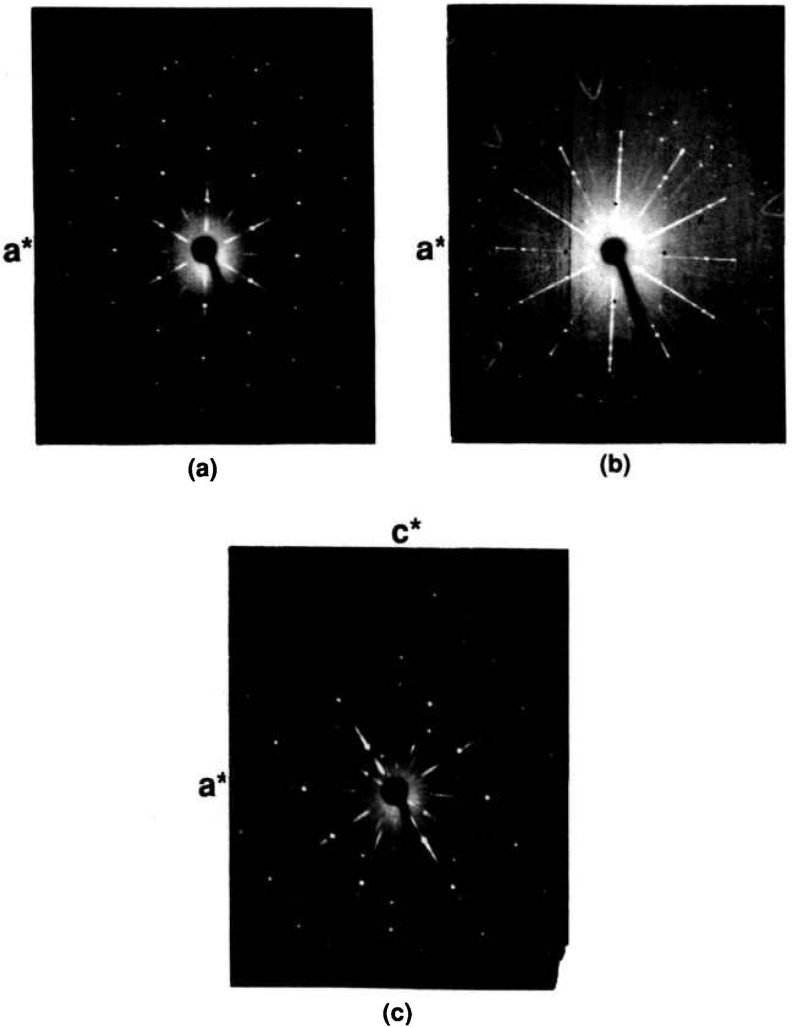
X-ray precession photographs of Sr_3_Bi_2_O_6_ (a) *hk0*, (b) unscreened *hk0* and (c) *h0l.*

**Figure 10:**
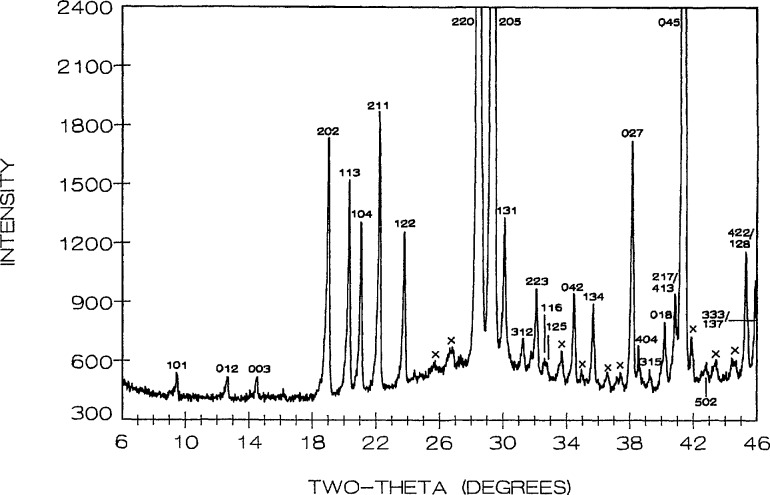
X-ray powder diffraction pattern of Sr_3_Bi_2_O_6_ (cooled from 975 °C). X=unidentified peaks-probably due to hydration.

Sr_3_Bi_2_O_6_ melts incongruently between 1200 and 1220 °C. Single crystals are formed in many compositions in the ternary system with CuO when heated above ~900 °C. Apparently, this phase has a large primary phase field in the ternary system. For example, single crystals were obtained from $\mathrm{SrO}:\frac{1}{2}{\mathrm{Bi}}_{2}O_{3}:\mathrm{CuO}$ 55:35:10 at 900 °C and from $\mathrm{SrO}:\frac{1}{2}{\mathrm{Bi}}_{2}O_{3}$ 57.5:42.5 at 1000 °C. These crystals often react slowly with atmospheric moisture. The best crystals were obtained using an NaCl:KCl flux with 4/1 flux/Sr_6_Bi_2_O_9_ ratio cooled from 1025 to 650 °C at 5 °/h (table 1b). These crystals are colorless and easily recognized because of their very low birefringence in polarized light. All these crystals were found (see precession photographs, fig. 9) to be rhombohedral probably $R\bar{3}m$, with unit cell dimensions refined from the x-ray diffraction powder data (table 6, fig. 10) *a* = 12.526(1), *c* = 18.331(2) Å.

#### 3.3.6 Sr_6_Bi_2_O_9_(S_3_B)

**Figure 11:**
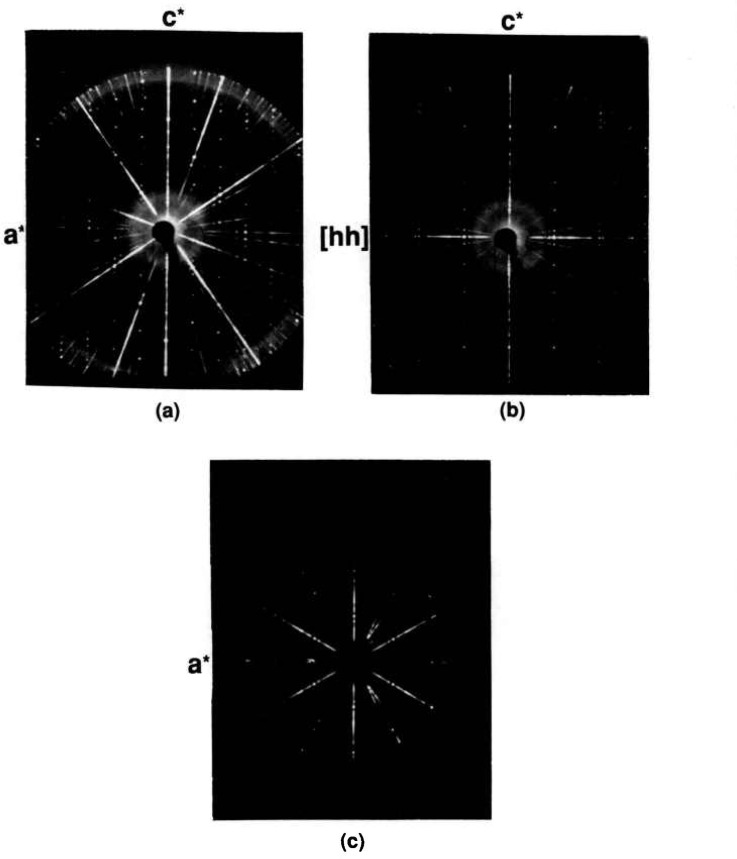
X-ray precession photograph of “Sr_6_Bi_2_O_9_” (a) *h0l*, (b) *hhl* and (c) unscreened *hk0.*

**Figure 12:**
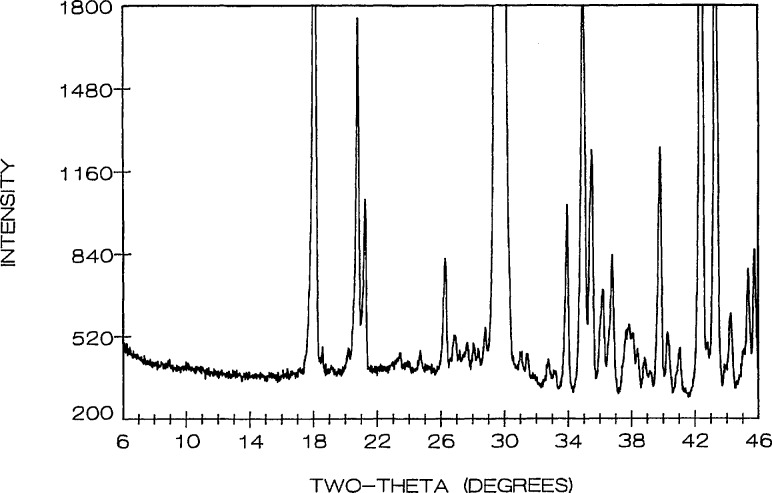
X-ray powder diffraction pattern of Sr_6_Bi_2_O_9_ (heated to 975 °C then cooled to 900 °C, held for 24 h and cooled to room temperature).

Previous workers [27] did not report any binary compound with more than 60 mole percent SrO; however, Sr_6_Bi_2_O_9_ appears to be stable between about 750 and 950 °C, and it decomposes between 950 and 975 *°*C to Sr_3_Bi_2_O_6_+SrO. Single crystals were obtained by heating a prereacted specimen plus 1:1 NaCl:KCl flux (flux/charge ratio =10/90). X-ray precession photographs (fig. 11) indicate an apparently rhombohedral unit cell with *a* =6.009 and *c* = 58.633 Å. This appears, however, to be a subcell and even a doubled *a*-axis (as suggested by electron diffraction data) does not account for all of the diffraction maxima observed in an x-ray powder diffraction pattern of the prereacted mix (table 7, fig. 12). The crystals may actually be an oxychloride phase and the pseudocell suggested in table 7 does not fit the observed data very accurately. The reaction Sr_6_Bi_2_O_9_→(975 °C)→Sr_3_Bi_2_O_6_+3SrO is completely reversible i.e., with material that was decomposed, Sr_6_Bi_2_O_9_→Sr_3_Bi_2_O_6_+3SrO at 975 °C, one can perform the back reaction, Sr_3_Bi_2_O_6_+3SrO→(900 °C)→Sr_6_Bi_2_O_9_, with or without intermediate grinding (and exposure to atmospheric CO_2_).

### 3.4 The System $\mathrm{SrO}:\frac{1}{2}{\mathrm{Bi}}_{2}O_{3}:\mathrm{CuO}$

**Figure 13:**
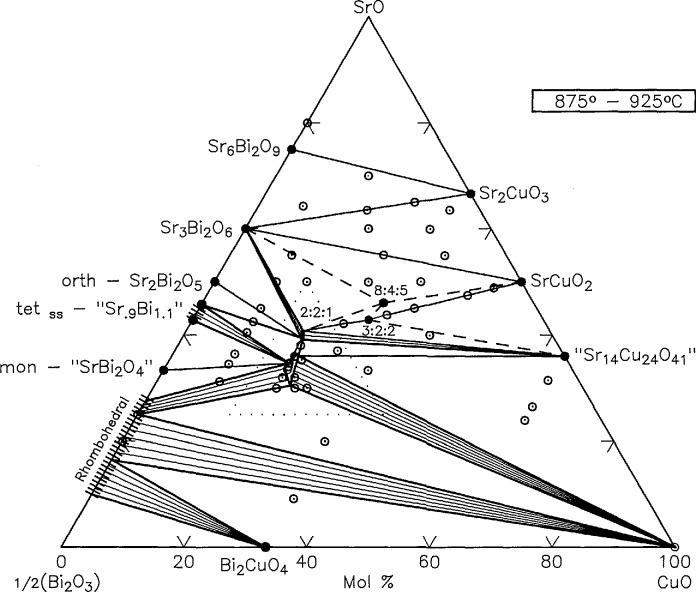
Phase diagram for the system $\mathrm{SrO}-\frac{1}{2}{\mathrm{Bi}}_{2}O_{3}-\mathrm{CuO}$○-compositions studied, ●-compounds. This diagram represents subsolidus conditions, although Bi_2_O_3_ melts at 825 °C and therefore partial melting occurs below 875 °C in most compositions below the join CuO-Rhomb. In addition, some melting was found at 875 °C for the composition 34.66:55.33:10.

**Figure 14:**
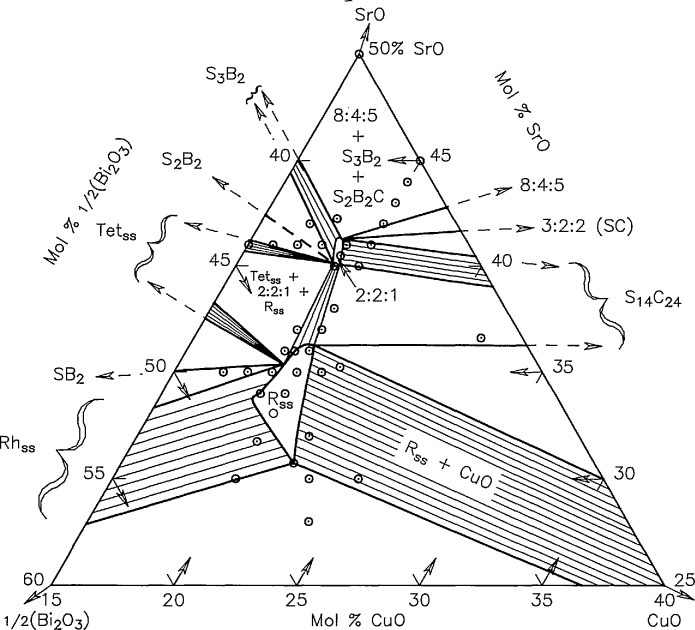
An enlargement of the triangular region of the phase diagram in figure 13 that is delineated by dots.

Phase relations in the nominally ternary system are shown in figure 13 and experimental data are reported in table 1. Figure 14 is an enlargement of the triangular region of figure 13 that is delineated by dots. Many of the experiments listed in table 1 yield apparently conflicting and often confusing results, precisely because the experimental system is not strictly ternary in air and/or in contact with various capsule materials such as Au, Pt or 70Ag30Pd. Reproducibility of experiments in this system is exceedingly difficult to achieve, and it is often impossible to reproduce the results published by others. In some cases this may be because msufficient experimental details were given; however, attempts to reproduce our own experiments have sometimes lead to slightly different results. Experimental results are greatly affected by the factors outlined below.

1. Compositional changes caused by reaction with Au or other containers;
2. Volatilization of Bi_2_O_3_;
3. CO_2_ in some phases at the lower temperatures (e.g., SrCO_3_ does not decompose in air until about 875 °C);
4. Oxidation/reduction reactions involving atmospheric O_2_, CO_2_, or H_2_O;
5. Difficulties related to the very disparate melting behaviors of various compounds and the end members. For example, Bi_2_O_3_ melts at ~825 °C but CuO decomposes in air to form Cu_2_O at about 1020 °C which melts at about 1210 °C. Also, The Sr-cuprates react very slowly at temperatures below the melting points of Bi_2_O_3_ and Bi_2_CuO_4_. Thus, it was often necessary to prepare specimens from prereacted compounds (or mixtures of compounds) instead of the end members.
6. Persistence of apparently unstable three phase assemblages within single phase regions. Typically, it is not possible to homogenize single phase ternary samples to the point that all detectable traces of additional phases are eliminated from x-ray powder patterns.

Therefore, it should be emphasized that the diagram in figure 13 is a composite of subsolidus data that is neither strictly ternary nor strictly isothermal. The region below the join that connects CuO to the SrO-poor end of the rhombohedral Sillen phase field contains phases which melt below 850 °C, and some phases in the low CuO portion of the system begin to melt between ~875 and 900 °C. Also, specimens of one composition which are near the SrBi_2_O_4_:Raveau-solid-solution join showed evidence of melting between 850 and 875 °C. All other compositions start to melt above at least 900 °C and many start melting slightly above 925 °C.

#### 3.4.1 Sr_2_Bi_2_CuO_6_(S_2_B_2_C-2:2:l)

This compound should nominally be the end member with *n* = 1 of the homologous series Sr_2_Bi_2_Ca*_n_*_−1_Cu*_n_*O_2_*_n_*_+4_. However, the x-ray powder diffraction pattern for this composition does not match at all with the predicted tetragonal subcell for a compound of this structure type. The predicted type of x-ray pattern is only found in specimens that are grossly deficient in SrO (i.e., compositions corresponding to the Raveau solid solution region—see below). The compound which occurs at approximately Sr_2_Bi_2_CuO_6_ has been characterized by electron diffraction and x-ray powder and single crystal diffraction and the results reported elsewhere [18]. The compound was found to be monoclinic, space group C2/m (or Cm) with *a* =24.493(2), *b* = 5.4223(5), *c*=21.959(2) Å, *β*=105.40(1)°. The actual composition with Sr:Bi:Cu ratio of 2:2:1 always contains a small amount of Sr_14_Cu_24_O_41_ and probably also some of the Raveau-type phase. Therefore, this compound is shown in figures 13 and 14 as being slightly deficient in CuO (less than 1 mol percent) and having a small homogeneity region. The x-ray powder diffraction data, single crystal precession photographs and electron microscopy data, along with figure 14, were previously published [18]. This phase appears to have a subcell with *c*-subcell (~5.49 Å) $\frac{1}{4}c$-supercell; electron microscopy data for some grains indicate an incommensurate superstructure. The x-ray diffraction data for compositions with only 19 mol percent CuO do not yield satisfactory least-squares refinements. It is possible that the observed incommensurate modulation is an equilibrium phenomenon dependent on composition, although it is equally likely to be due to a non-equilibrium chemical inhomogeneity.

#### 3.4.2 The Raveau-Type Solid Solution (Rav)

A two-phase region is shown in figure 14 (after [18]) between the 2:2:1 phase and the region referred to as the Raveau-type solid solution. This nomenclature is used because, structurally, the Raveau-type phase most closely resembles the *n* = 1 end member of the series Sr_2_Bi_2_Ca*_n_*_-1_Cu*_n_*O_2_*_n_*_+4_ and because Raveau and coworkers were the first to report superconductivity in this system [31]. This phase often forms metastably as an almost single-phase product when compositions near the indicated equilibrium single-phase region are synthesized by cooling from a melt. For example, a melt of 2:2:1 composition first crystallizes as the Raveau solid solution and reacts to form the 2:2:1 phase only after subsequent heating and grinding (table 1); similarly, when a mixture of composition 3:2:2 was prepared by a lactate route, the Raveau solid solution was the first crystalline phase to form; but, the 3:2:2 phase replaced it after subsequent heating and grinding (table 1). The crystals formed from melts of Raveau solid solution, or similar compositions (outside the equilibrium Raveau field), are always very platy and micaceous and form “books” of crystals not well ordered in the direction perpendicular to the plates. They always have one long crystallographic axis of about 26.6 Å and the x-ray powder diffraction data can be roughly fit to a pseudotetragonal subcell with *a* =5.3 Å. Several unit cells have been reported for this phase, either pseudotetragonal or pseudoorthorhombic [32,33].

**Figure 15:**
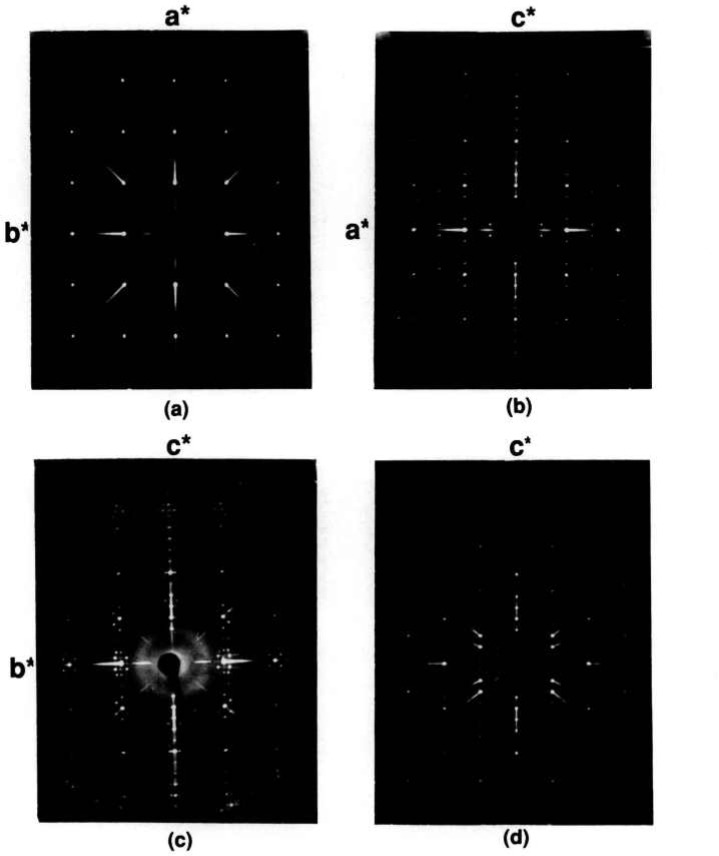
X-ray precession photographs of an orthorhorabic/incommensurate Raveau solid solution phase that was grown in 1:1 NaF:KF flux. Original composition=Sr_2_Bi_2_CuO_6_ (a) *hk0*, (b) *h0l*, (c) *0kl* and (d) *hhl.*

**Figure 16:**
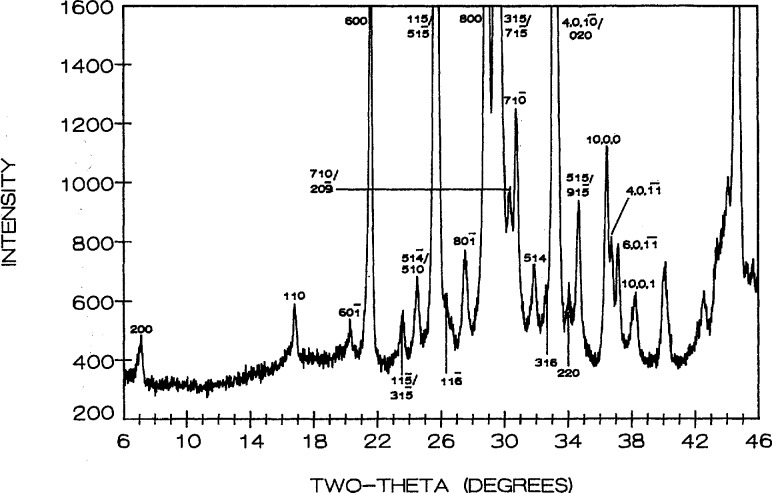
X-ray powder diffraction pattern of the Raveau phase from the composition Sr_9_Bi_11_Cu_5_O_30.5±_*_x_* (cooled from 875 °C).

Crystals that were picked from various ternary melts (with or without chloride flux) were invariably non-single and appear to have a monoclinic superstructure. The phase formed using 1:1 NaF:KF flux, however, yielded crystals with apparent orthorhombic symmetry and a very strange incommensurate superstructure (fig. 15). Onoda and Sato [34] obtained a monoclinic superstructure for a crystal that was grown from a melt of 1:1:1 composition (Sr:Bi:Cu= 1:1:1) which was heated in an Al_2_O_3_ crucible. They report a nominal composition for the crystal of Sr:Bi:Cu 4:6:3, well outside the equilibrium single-phase region reported in figures 13 and 14. The unit cell reported for this phase [34] is C-centered monoclinic with *a* =26.856, *b*=5.380, *c* = 26.908 Å, *β* = 113.55°; no data were reported on the extent of contamination from the Al_2_O_3_ crucible. A calculated powder pattern based on their structure determination [34] was obtained from M. Onoda (private communication) and these data were used to index the x-ray powder diffraction pattern of the composition with Sr:Bi:Cu ratios of 36:44:20 (near the SrO-rich end of the Raveau solid solution region). All of the super-structure lines observed for this composition can be completely accounted for by *hkl’s* with intensities *very* similar to those calculated by Onoda. For a C-centered monoclinic cell, the unit cell dimensions obtained by least-squares analysis of this x-ray powder data (table 8, fig. 16) are *a* =26.889(9), *b* = 5.384(2), *c*=26.933(8) Å, *β*= 113.67(3)°.

It should be noted, however, that powder patterns for more Bi-rich Raveau-type solid solutions display superstructure peaks which deviate widely from those observed for the 36:44:20 composition. At present it is not known if this is truly a region of solid solution or a collection of smaller regions (separated by two and/or three phase fields) in which several structurally related phases are stable. New specimens are currently being prepared at very close intervals in this Raveau-type region in order to determine the true crystal chemistry of this important “phase.” These results will be reported in the near future [35].

The Raveau solid solution region extends along a line with approximately 20 mol percent CuO according to the formula Sr_1.8−_*_x_*Bi_2.2+_*_x_*CuO*_z_* with ~0.0< *x* < ~0.15. This is slightly at odds with the results of Saggio et al. [36] who reported the formula Sr_1.8+_*_x_*Bi_2.2−_*_x_*CuO*_z_* with 0.0 < *x* <0.08 which corresponds to negative values of *x* in our formula. Their samples were annealed at 800 °C and premixed with 0.5 weight percent Li_2_CO_3_. It is not known if the differences between their results and ours are due to the temperature difference, the time of “equilibration,” or to the presence of Li_2_CO_3_. They also report [36] that only the high SrO end of the solid solution exhibits superconductivity based on the data of Akimitsu et al. [37] which were obtained from specimens that were heated twice at 880 °C for 12 h. This preparation should probably have yielded results similar to ours, but we failed to find evidence of superconductivity at temperatures above 10 K. It is possible that superconductivity only occurs in metastable Raveau-type solutions that have compositions which lie outside the equilibrium “single phase” field.

The Raveau-type solid solution also exhibits non-stoichiometry with respect to its CuO concentration. The solid solution region corresponds approximately to the formula Sr_1.8−_*_x_*Bi_2.2+_*_x_*Cu_1±_*_x_*_/2_O*_z_*. Of course, there is no *a priori* reason why the CuO concentration must be structurally controlled by the Sr/Bi ratio.

Chakoumakos et al. [38] reported the results of a study of Raveau-type single crystals that were grown under oxygen from CuO-rich melts in crucibles of various compositions. Incomensurate superstructure peaks (related to orthorhombic symmetry) were found to vary systematically with the SrO content. Superconductivity was found to be related to excess oxygen and to the concentration of impurities including Al_2_O_3_. The superstructure peaks occurred with modulation of ~ 1/5b* plus a c* component varying from 0.29c* to 0.65c* (where * represents the reciprocal vector direction). The observed formula for these crystals was reported as Bi_2_Sr_2−_*_x_*CuO_6−_*_y_*. These crystals (and most if not all melt-grown, Raveau-type crystals) are probably metastable since they have compositions well outside the equilibrium range shown in figures 13 and 14. It should be noted, however, that Chakoumakos et al. grew their crystals under oxygen rather than air, so the relevant single-phase region may be similar but will not be identical to that in figures 13 and 14.

#### 3.4.3 Sr_8_Bi_4_Cu_5_O_19+_*_x_* (S_8_B_4_C_5_-8:4:5)

**Figure 17:**
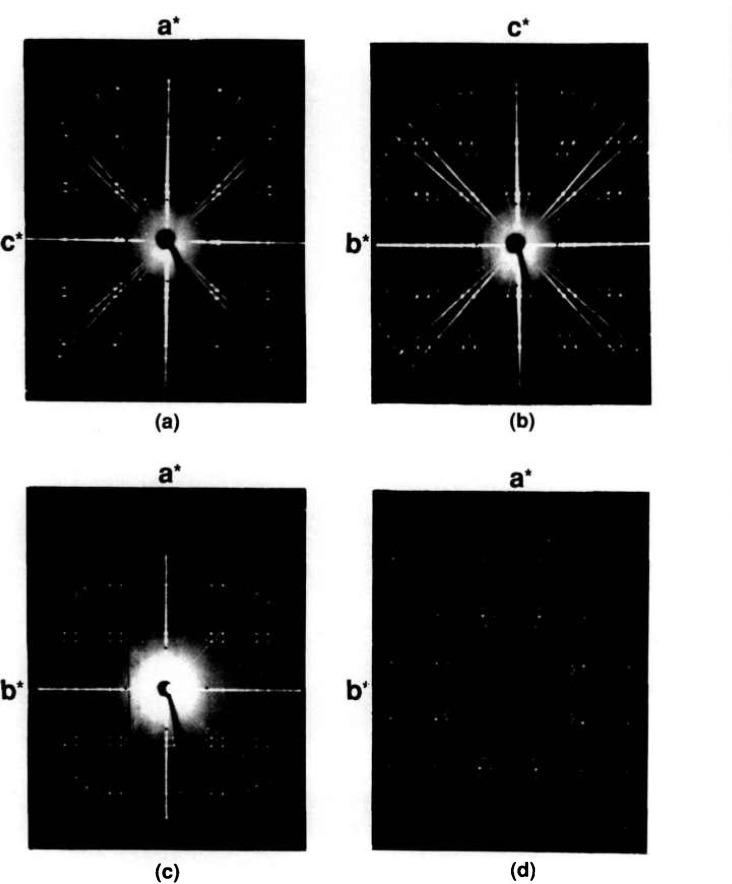
X-ray precession photographs of 8:4:5 (a) *h0l*, (b) *0kl*, (c) *hk0* and (d) *hkl.*

This phase was apparently first described [39] as a compound with the composition Sr_4_Bi_2_CuO_9+_*_z_* (Sr:Bi:Cu=2:1:1); however, an examination of the reported unindexed x-ray powder diffraction data indicate that modest amounts of both S_3_B_2_ and SC were present in this sample. All of our experiments with the 2:1:1 composition yielded three phases when equilibrated in air at subsolidus temperatures, although the minority phases that were observed depended upon the heat treatment (table 1). Small single crystals of this new phase were obtained from a specimen of 2:1:1 that was mixed with 10 weight percent 1:1 NaCl:KCl flux and sealed in a gold tube that was heated at 900 °C for 1 h then cooled to 650 °C at 3 °C/h. The crystals are needle-like suggesting that one crystallographic axis is probably much shorter than the others, and x-ray precession photographs (fig. 17) revealed that it is orthorhombic (space group Fmmm) with *a, b, c* parameters of approximately 33.98, 24.02, 5.364 Å, respectively. The crystal structure of this phase has been solved by Fuertes et al. [40] who describe its chemistry as Bi_4_Sr_8_Cu_5_O_19+_*_x_* and its unit cell as orthorhombic with *a* =5.373(2), *b*=33.907(6), *c* =23.966(4) Å. Obviously, the diffraction data in figure 17 indicate that this is the same phase as the one reported in [39,40].

**Figure 18:**
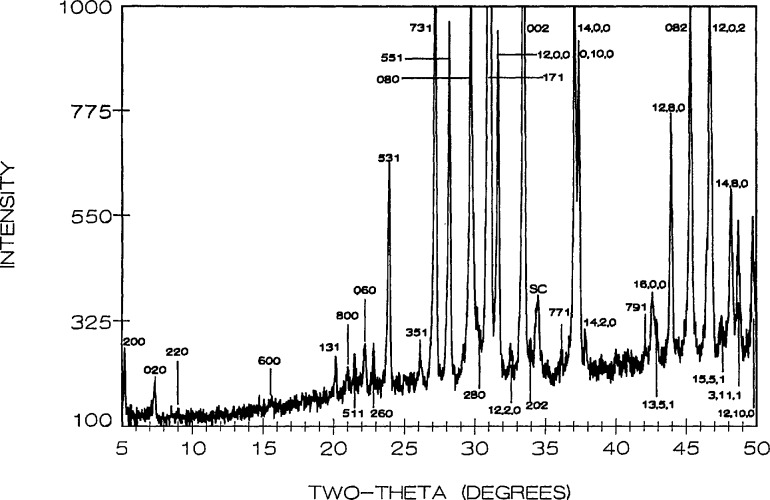
X-ray powder diffraction pattern of Sr_8_Bi_4_Cu_5_O_19+_*_x_* (cooled from 925 °C in O_2_).

Single-phase specimens of Sr_8_Bi_4_Cu_5_O_19+_*_x_* were only obtained in this laboratory when the starting materials were annealed in one atmosphere of oxygen. The unit cell refined from the data obtained from the 8:4:5 specimen (table 9, fig. 18) is orthorhombic Fmmm with *a* =33.991(3), *b*=24.095(2), *c*=5.3677(5). Clearly the published structure of this phase [40] requires more than the 19 oxygen atoms per formula unit that are implied by an 8:4:5 ratio. The smaller unit cell obtained by [40] was also found in the present work when an 8:4:5 specimen was melted in an Al_2_O_3_ crucible (as were the crystals reported by [40]) poured onto an Al plate and annealed in air or oxygen. Attempts to supply the excess oxygen by the substitution of some La^+3^ for some of the Sr^+2^ as suggested by R. J. Cava (private communication) was only partially successful, never resulting in a completely single-phase specimen when heated in air.

#### 3.4.4 Sr_3_Bi_2_Cu_2_O_8_ (S_3_B_2_C_2_-3:2:2)

Extrapolation based on the general formula for the homologous series of Bi-containing high-*T*_c_ phases, A_2_Ca*_n_*_−1_B_2_Cu*_n_*O_2_*_n_*_+4_, predicts the formula Sr_2_CaBi_2_Cu_2_O_8_ (2:1:2:2) for the phase with *n*=2, and a *c*-axis of ~30.6 Å which implies *d*(002) ~5.78 ° 2*θ* for Cu*K α* radiation. It is known that Sr^+2^ can substitute for some of the Ca^+2^ up to at least 3:3:4:4 [40]. If *all* the Ca^+2^ were replaced by Sr^+2^, the chemical formula would degenerate to 3:2:2 or Sr_3_Bi_2_Cu_2_O_8_; but, attempts to synthesis the *n* = 2 phase at this composition have failed. The presence of a small peak at ~5.75° 2*θ* was noted during the first low temperature calcination of specimens prepared by decomposition of lactate precursor powders with 3:2:2 composition. However, the peak at ~5.75° 2*θ* disappears after subsequent heat treatments which suggests that it is associated with a metastable phase.

**Figure 19:**
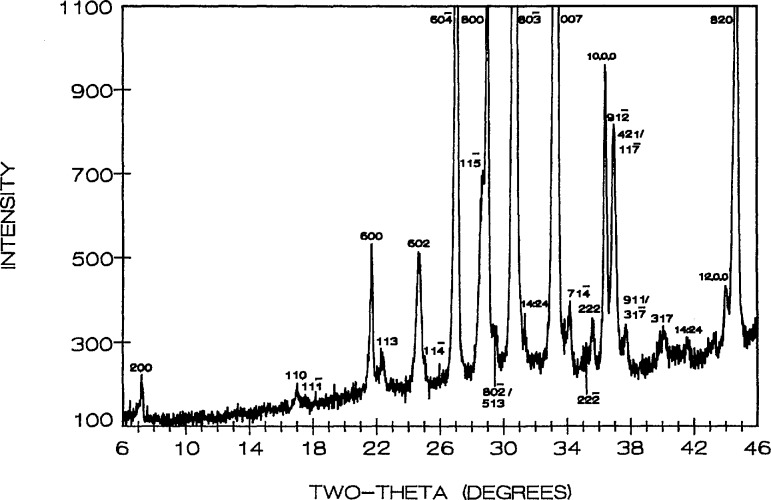
X-ray powder diffraction pattern of Sr3Bi2Cu20s (cooled from 925 °C in O2).

Compositions of 3:2:2 prepared by conventional solid state techniques yield a new phase that has an x-ray powder diffraction pattern (table 10, fig. 19) which resembles both the Raveau-type solid solution and the 2:2:1 phase in some respects. The low angle peak occurs at about the same value as for the Raveau solid solution (*d ~* 12.35 Å, 2*θ* ~7.15°), but there is a very small peak at a *d*-value of twice that (*d*~24.7 Å, 2*θ* ~3.58°). The strong (113) Raveau-type tetragonal subcell peak at ~25.75° 2*θ* is not present and, instead, a strong peak occurs at ~26.85° 2*θ*, similar to the 2:2:1 compound. In addition, there are considerable differences between this pattern and both the Raveau solid solution and Sr_2_Bi_2_CuO_6_, which indicate that Sr_3_Bi_2_Cu_2_O_8_ is a unique phase. As yet, no single crystals of this phase have been synthesized. The pattern in figure 19 shows the presence of a small amount of Sr_14_Cu_24_O_41_, indicating some probable nonstoichiometry in the composition. The diffraction maxima in this pattern have been indexed with comparison to the 2:2:1 and Raveau solid solution with a C-centered monoclinic unit cell, *a* =24.937(7), *b* = 5.395(2), *c* = 19.094(7) Å, and *β*=96.97(3)°. This commensurate cell probably represents only a subcell of an incommensurate non-stoichiometric phase.

#### 3.4.5 Miscellaneous Phases of Unknown Composition

Two phases high in SrO content at approximate Sr:Bi:Cu ratios of 9:4:1 and 7:2:2 were reported by Saggio et al. [36], and two different phases at 4:2:1 and 2:1:1 were reported by Casais et al. [39]. Of these, we only found evidence for the phase reported at 7:2:2 composition, and then only at temperatures below 875 °C. The Saggio et al. data [36] are complicated by their use of the 0.5 wt% Li_2_CO_3_ “as a mineralizer.” Peaks corresponding to the *d*-spacings reported for the composition 9:4:1 were not present in our specimens except when we included 0.5 wt% Li_2_CO_3_, and the binary phase Sr_6_Bi_2_O_9_ (that was not reported by Saggio et al. [36]) is only present when Li_2_CO_3_ is absent. We therefore conclude that the “9:4:1-phase” is not present in the ternary system. Some of the low-angle *d*-spacings reported for the “7:2:2-phase” (4.82 Å=18.40° 2*θ* and 4.17 Å=21.27° 2*θ*) in samples that were heated at 800 °C were observed in patterns from samples that we heated at temperatures below ~875 °C (table 1). Because SrCO_3_ does not decompose until ~ 875 °C, these results suggest the presence of one or more oxycarbonate phases. The first two *d*-spacings as well as the strongest peak reported as a “4:2:1” phase [38] (*d*=4.91, 4.25 and 3.004 Å) are apparently due to the phase Sr_6_Bi_2_O_9_(S_3_B).

In summary, we interpret the evidence for these four reported phases as follows:

9:4:1-mostly due to reaction with Li_2_CO_3_;
7:2:2-multiphase due to reaction with Li_2_CO_3_ plus a Sr:Bi:Cu-oxycarbonate;
4:2:1-Sr_6_Bi_2_O_9_+other phases; and
2:1:1-Sr_8_Bi_4_Cu_5_O_19+_*_x_*+S_3_B_2_+SC

On heating above about 850 °C, the diffraction maxima characterizing the 7:2:2 “phase” start to disappear and are ultimately replaced by at least one other strong maximum at ~ 30.25° 2*θ* the origin of which is still unknown. At the 3:1:1 composition (table 1) the 7:2:2-type phase is very prevalent at 750 and 800 °C; however, as it starts to decompose at 850 °C, another peak arises at ~ 11.00° 2*θ* which persists even at 900 °C after the first heat treatment but finally disappears after three overnight anneals. The origin of this ~ II.00° peak is also unknown but it appears to indicate a metastable phase that forms during decarbonation and subsequently decomposes.

At the 2:1:1 and 8:4:5 compositions it was found that preliminary low-temperature annealing was actually detrimental to the formation of an equilibrium assemblage. Apparently, an oxycarbonate phase characterized by small peaks at 2*θ* =4.40° and 5.60° with strong peaks at 30.50° and 32.45° is formed first with repeated heating at 750°C; further heat treatments at 800 °C produce a new peak at ~4.80° as the 4.40° peak gradually disappears. These are gradually replaced by peaks from the 2:2:1 and Raveau solid solution plus SrCuO_2_, but the 8:4:5 phase which should form is not found. Note, however, that when this sample was put in an Al_2_O_3_ crucible, melted and reheated at 900 °C, the 8:4:5 phase did form. Apparently, the formation of these oxycarbonates blocks the nucleation of 8:4:5.

Four ternary phases were reported in this system by Ikeda et al. [42]. These are essentially the same phases as those reported here, although the compositions do not always agree. The formula given for the Raveau phase solid solution differs somewhat from that used here. The formula for Sr_2_Bi_2_CuO_6_ is given as Sr_16_B_17_Cu_7_O*_z_*, considerably deficient in SrO and occurring in the region clearly shown by our work to contain three phases. The x-ray diffraction pattern shown for their Sr_3_Bi_2_Cu_2_O*_z_* clearly shows evidence of the Sr_14_Cu_24_O_41_ phase, as do our own patterns of this composition. Unit cell dimensions and symmetry given by Ikeda et al. [42] and Saggio et al. [36] for their ternary phases are clearly based on intuition rather than single crystal data and should be considered suspect.

#### 3.4.6 Deduction of Ternary Compatibility (Alke-made) Lines

This ternary system is remarkable for the gross irreproducibility of the experimental results. Attainment of equilibrium for each of the ternary compounds that we represent as stable is very difficult and time consuming. Nevertheless, equilibrium can generally be more easily achieved in ternary combinations furthest from the compositions of the stable ternary phases. For this reason the deduction of the compatibility joins is somewhat more reliable than one might suppose based on the difficulties inherent in determining the true compositions of the ternary phases.

Some generalizations can be made concerning both the data in table 1 and the interpretations behind our construction of figures 13 and 14. Because there is a two phase region involving CuO and the rhombohedral Sillen-phase solid solution, the compound Bi_2_CuO_4_ and the low melting eutectics of the Bi_2_O_3_-CuO binary system are not involved in most of the ternary equilibria. Also, CuO is in equilibrium with most or all of the compositions comprising the Raveau-type solid solution region. Therefore, the 1:1:1 composition (reported by Raveau [31] as superconducting) is in the middle of a ternary phase field bounded by CuO, Raveau solid solution and S_14_C_24_. The compound Sr_14_Cu_24_O_41_ is in equilibrium with all three of the ternary phases related to the structurally homologous series A_2_Ca*_n_*_−1_B_2_Cu*_n_*O_2_*_n_*_+4_: Sr_2_Bi_2_CuO_6_, Sr_3_Bi_2_Cu_2_O_8_ and Sr_1.8−_*_x_*Bi_2.2+_*_x_*Cu_1±_*_x_*_/2_O*_z_* (i.e., 2:2:1, 3:2:2 and the Raveau solid solution), but not with the structurally dissimilar phase Sr_8_Bi_4_Cu_5_O_19+_*_z_* (8:4:5) or any of the SrO-Bi_2_O_3_ binary phases. The compound SrCuO_2_ is in equilibrium with all three of the ternary compounds except for the Raveau-type solid solution while Sr_2_CuO_3_ is compatible only with the two high SrO content binary phases but not with any of the ternary phases. Joins describing compatibility conditions for the 8:4:5 and 3:2:2 phases are left as dashed lines because of the difficulty in determining equilibrium three phase assemblages.

## Figures and Tables

**Figure 4a:**
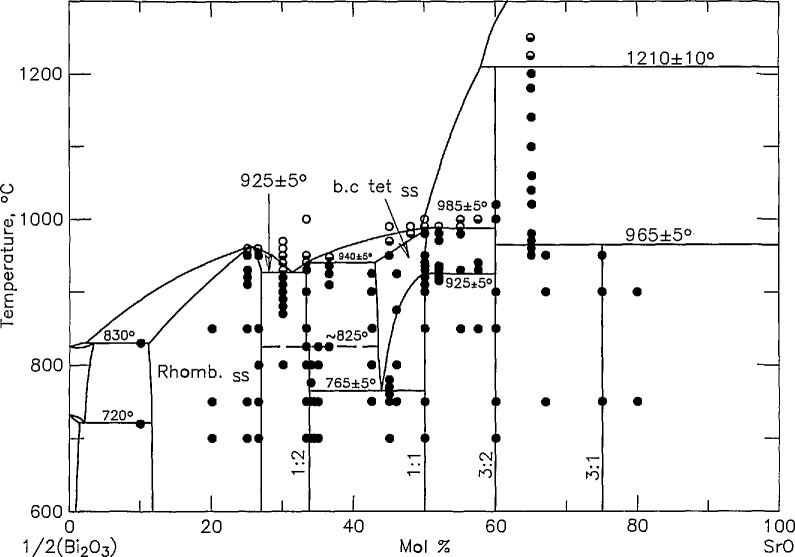
Phase diagram for the system $\mathrm{SrO}-\frac{1}{2}{\mathrm{Bi}}_{2}O_{3}$ as reported in [28] ●-not melted, ◒-partially melted, ○-completely melted.

**Figure 4b:**
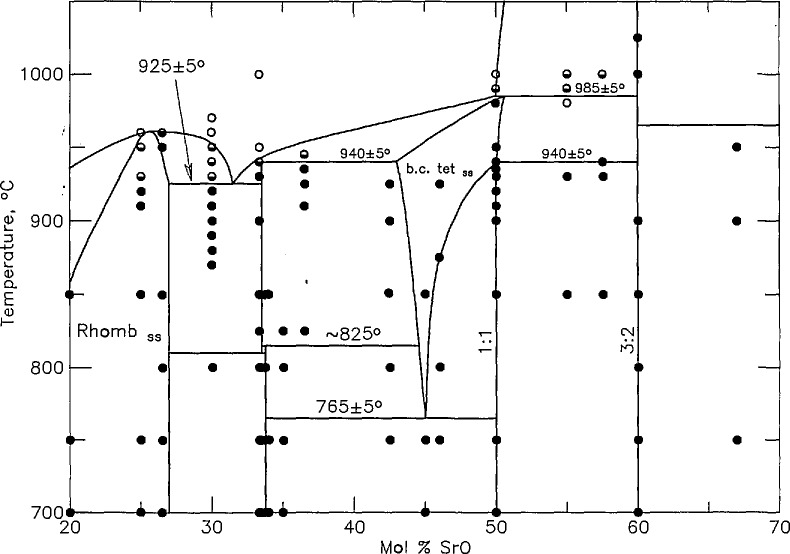
Enlargement of figure 4a showing polymorphism of SrBi_2_O_4_.

**Table 1a:** Experimental data for the ternary system SrO-Bi_2_O_3_-CuO

Spec.	Composition, mole percent^a^			Temperature of heat treatment; °C^b^		Visual observation	Results of x-ray diffraction^c^
no.	SrO	$\frac{1}{2}{\mathrm{Bi}}_{2}O_{3}$	CuO	Initial	Final		
	75.0	12.5	12.5	700			
				750			SrC0_3_+S_3_B+ “7:2:2”
				800			S_3_B+SrCO_3_ + S_2_C+ “7:2:2”_tr_
				850			S_3_B+S_2_C(+SrO?)
					900		S_3_B + S_2_C(+SrO?)
	65	10	25	700			
				750			SrC0_3_+CuO+ “7:2:2”+S_14_C_24_+S_3_B_tr_
				800			“7:2:2”+S_2_C+S_3_B+CuO_tr_
				850			S_2_C + S_3_B+“7:2:2”
				900			S_2_C+SC+S_3_B_2tr_+“7:2:2”_tr_
	64.29	28.57	7.14				
	*SrCO_3_:S_2_C:S_3_B_2_*		800×3			S_3_B+S_3_B_2_+“7:2:2”	
	1: 1 :2			800×5			S_3_B+S_3_B_2_+“7:2:2”
#1^d^	63.63	18.18	18.18	700			
				750			
				800			“7:2:2”+S_3_B+S_3_B_2_+S_2_C+SC+CuO
				800×3			“7:2:2”+S_3_B+S_3_B_2_+SC+S_2_C
				800×6			“7:2:2” + S_3_B+S_3_B_2_+SC+S_2_C
				850			S_3_B_2_+S_2_C + “7:2:2”+SC+S_3_B
#2	*S_2_C:S_3_B_2_*				875×1		S_3_B_2_ + S_2_C+X(30.25°)
	2:1				875×2		S_3_B_2_+S_2_C+X(30.25°)
					875×4		S_3_B_2_+S_2_C+X(30.25°)_tr_
#3	*S_2_d:S_3_B_2_*			800×3			S_3_B+S_3_B_2_+ “7:2:2”+S_2_C+SC
	2:1			800×5			S_3_B+“7:2:2” + SC + S_2_C_tr_+S_3_B_2tr_
					900×3		S_3_B_2_+S_2_C+S_3_B_tr_+X(30.25°)_tr_
#1	63.33	5.00	31.67				
	$\frac{1}{2}$*Bi_2_O_3_:S_2_C*		750				
	1.00:6.33		850				
					900		S_2_C+SC+S_3_B_2_+X_tr_
					950		S_2_C + SC+S_3_B_2_+X_tr_
#2	$\frac{1}{2}$*Bi_2_O_3_:S_2_C*			875×5		S_2_C+SC+S_3_B_2_+X	
	1.00:6.33						
#1	60	10	30				
	$\frac{1}{2}$*Bi_2_O_3_:S_2_C*			750			
	1:3			850	900		S_2_C+SC+S_3_B_2_+X_tr_
					950		S_2_C+SC+S_3_B_2_+X_tr_
#2	$\frac{1}{2}$*Bi_2_O_3_:S_2_C*			875×5		S_2_C+SC + S_3_B_2_+X	
	1:3						
#1	60	20	20	700			
				750			“7:2:2” + S_3_B+CuO+SrCO_3_
				800			“7:2:2” + SC+S_2_C
				850			SC+S_2_C+unk(11°)+“7:2:2”
					900		SC+S_2_C+unk(11°)
					900×3		S_3_B_2_+SC + S_2_C
#2				700			
				750			
				800			“7:2:2”+S_3_B_2_+SC+S_2_C+S_3_B+CuO
				800×3			“7:2:2”+S_3_B_2_+SC+S_2_C+S_3_B
					800×6		“7:2:2”+S_3_B_2_+SC+S_2_C+S_3_B
					850		S_3_B_2_+S_2_C+SC+“7:2:2”
#3	*S_2_C:S_2_B_2_*			700			
	2:1			750			
				800			S_3_B_2_+SC+“7:2:2”+S_2_C+S_3_B
				800×3			S_3_B_2_+SC+“7:2:2”+S_2_C+S_3_B
					800×6		“7:2:2”+S_3_B_2_+SC+S2C+S_3_B
					850		S_3_B_2_+S_2_C+SC+“7:2:2”
	57.14	28.57	14.29	700			
				850	875		S_3_B_2_+SC+2:2:1_tr_
					900		S_3_B_2_+SC+2:2:1_tr_
					900×3		S_3_B_2_+SC+2:2:l_tr_
	55	35	10		875(Ag/Pd^e^)		S_3_B_2_+2:2:1+X
					900(Ag/Pd^e^)		S_3_B_2_+2:2:1+X
	55	20	25				
	*SC:S_3_B_2_*				875		SC+S_3_B_2_+8:4:5
	2.5:1.0				875×2		SC+S_3_B_2_+8:4:5
					875×4		sc+s_3_b_2_+8:4:5_tr_
#1	55	10	35				
	$\frac{1}{2}$*Bi_2_O_3_:S_2_C:SC*		750				
	2:4:3			850			
					900		SC+S_2_C+S_3_B_2_
					950		SC+S_2_C+S_3_B_2_
#2	$\frac{1}{2}$*Bi_2_O_3_:S_2_C:SC*			875×5		SC+SiC+S_3_B_2_+X	
	2:4:3						
	50	40	10	850			S_3_B_2_+2:2:1
					875		S_3_B_2_+2:2:1
#1	50	35	15	875			S_3_B_2_+2:2:1
					900		S_3_B_2_2:2:1+8:4:5+SC_tr_
					900-3days		S_3_B_2_+2:2:l+3:2:2+8:4:5+SC
					900×3		S_3_B_2_+2:2:l + 8:4:5+3:2:2+SC
#2	*S_2_B_2_:SC*			650			
	1.1667:1.0000		750				
				800			2:2:1+S_3_B_2_+SC
					875		2:2:1 +S_3_B_2_+SC
#1	50	25	25	700			
				750×2			SrCO_3_+CuO+S_3_B+ S_14_C_24_+ “7:2:2”
					750×4(Au^f^)		* + SC_tr_+ S_14_C_24tr_
					800(Au)		* + 8:4:5_tr_+SC_tr_+S_14_C_24tr_
					800×2(Au^f^)		* + 8:4:5_tr_+SC_tr_+S_14_C_24tr_
					850(Au^f^)		8:4:5+*+SC_tr_
					850×2(Au^f^)		8:4:5+S_3_B_2_+SC
					850×3(Au^f^)		8:4:5+S_3_B_2_+SC
					880×1(Au^f^)		8:4:5+S_3_B_2_+SC
					900(Au^f^)		8:4:5+S_3_B_2_+SC
#2	*SC:S_2_B_2_*			880×1			SC+2:2:1 + S_3_B_2_
	1.0:0.5			880×5			8:4:5+2:2:l + S_3_B_2_+SC
				900×3			8:4:5 + S_3_B_2_+SC
#3				650			
				750			
				800			S_3_B_2_+SC+2:2:1
					875		SC+2:2:l + S_3_B_2_+8:4:5
					900(Au^f^)		SC + 8:4:5+ S_3_B_2_
					900×3(Au^f^)		SC+8:4:5 + S_3_B_2_
					900×6(Au^f^)		SC+8:4:5 + S_3_B_2_
					925(Au^f^)		SC+8:4:5 + S_3_B_2_
					950(Au^f^)	part.melt	SC+S_3_B_2_+Rav
				950(Au^f^)			
					900(Au^f^)		SC+S_3_B_2_+8:4:5
					875(Au^f^)		SC+S_3_B_2_+8:4:5
	50.00	16.50	33.50	650			
				750			SrCO_3_ + CuO + “7:2:2” + SC_tr_
				800			CuO+SC + “7:2:2” + S_14_C_24_
				850			SC+S_3_B_2_+2:2:1 + S_2_C
					875		SC+S_3_B_2_+2:2:1
					900		SC+S_3_B_2_+2:2:1+ 8:4:5
					900×3		SC+S_3_B_2_ + 8:4:5
#1	48.75	5.00	46.25				
	*SC:SB_2_*			750			
	18.5:1.0			850			
					900		SC+2:2:1 + 8:4:5
					950	sl.melting	SC+Rav+S_3_B_2tr_
#2	*SC:SB_2_*						
	18.5:1.0				875×5		SC+8:4:5+X
#1	47.5	10.0	42.5				
	*SC:SB_2_*			750			
	8.5:1.0			850			
					900		SC+2:2:l + 3:2:2+8:4:5_tr_
					950	part.melt	SC+Rav + S_3_B_2tr_
#2	*SC:SB_2_*				875×5		SC+8:4:5 + 3:2:2
	8.5:1.0						
#1	47.06	23.53	29.41				
	(8:4:5)			700			
				750×2			SrC0_3_+CuO+Rav+unk(4.40°)
					800(Au^f^)		SrC0_3_+CuO + Rav+unk(4.40°) + unk(4.80°)
					850(Au^f^)		unk(4.80°) + CuO + SrCO_3_
					850×2(Au^f^)		unk(4.80°) + CuO + SrCO_3_
					875(Au^f^)		
					900(Au^f^)		2:2:1+ Rav+SC
				900(Au^f^)			unk(4.40°)+unk(4.80°)+CuO
#2				875			S_3_B_2_+2:2:l + SC+S_14_C_24_+Rav + 3:2:2+S_3_B
					900		S_3_B_2_ + SC+2:2:l + 3:2:2+8:4:5
					900×2		S_3_B_2_ + SC+2:2:l + 3:2:2+8:4:5
					950	part.melt	S_3_B_2_+Rav+SC
#3L^g^				650			B_2_C+SrCO_3_+CuO
				750			
				850			2:2:1+ S_3_B_2_+SC+3:2:2+S_14_C_24_
					850×2		2:2:1+ S_3_B_2_+SC+3:2:2+S_14_C_24_
				450			
				850×2			
					900×1		8:4:5+ 2:2:1 +SC
					900×4		8:4:5 + 2:2:1 + SC_tr_
					925		8:4:5+ SC_tr_
#4				850			
				1250^h^		comp.melt	
					900(O_2_^i^)		8:4:5
					925(O_2_^i^)		8:4:5
#1	45	20	35	850			
				875	875×7		SC+3:2:2+S_14_Cu_24_
					900		SC+Rav+S_3_B_2_+8:4:5
					900×3		SC+ 3:2:2
#2				875			
					900		3:2:2+SC+2:2:l
#3	*SC:SB_2_*			800			
	3.5:1.0			875×1			SC+S_14_C_24tr_
					875×6		SC+2:2:1+ 8:4:5
	45	45	10	700			
				800			
				850			
					875		S2B2 +2:2:1
	45	35	20	700			
				800			
				850			
					875		2:2:1 + S_3_B_2_+SC
					900		2:2:1 + S_3_B_2_+SC
	44.44	33.33	22.22	700			
				850			2:2:1 + S_3_B_2_+SC+S_14_C_24_
				875			2:2:l + S_3_B_2_+SC + S_14_C_24_+3:2:2_tr_
					900		2:2:l + S_3_B_2_+8:4:5 + 3:2:2+SC_tr_
					900×3		S_3_B_2_+Rav
	44	36	20	700			
				800			
				850			
					875		2:2:1 + S_3_B_2_ + SC
					900		2:2:1 + S_3_B_2_+SC
	43.75	25.00	31.25	700			
				750			
				850			
					875		3:2:2+SC+S_14_C_24_+S_3_B_2_
					900		3:2:2+SC+S_14_C_24_+2:2:l_tr_
					900×2		3:2:2+SC+S_14_C_24tr_
	43.62	32.98	23.40	700			
				750			
				850			
					875		2:2:1 +3:2:2+s_14_c_24_+sc
					900		2:2:l+3:2:2+SC+8:4:5_tr_
	43	37	20	700			
				800			
				850			
					875		2:2:1+SC+S_3_B_2_
					900		2:2:1+SC+S_3_B_2_
	42.86	32.65	24.49	700			
				750			
				850			
					875		2:2:1 + 3:2:2+S_14_C_24_+SC
					900		2:2:l + 3:2:2+S_14_C_24_+SC
#1	42.86	28.57	28.57	700			
	(3:2:2)			850			
				875			2:2:1+SC+S_14_C_24_+3:2:2+S_3_B_2tr_
					900×3(Au^f^)		2:2:1+SC+8:4:5+3:2:2+S_3_B_2tr_
					900×6(Au^f^)		2:2:1 + 8:4:5+S_3_B_2_
					900×8(Au^f^)		2:2:1 +8:4:5+S_3_B_2_
#2				700			
				750			
				850			
				875			2:2:1+SC+S_14_C_24_+3:2:2+S_3_B_2_
					900		3:2:2+SC_tr_+S_14_C_24tr_
					900×2		3:2:2+SC_tr_+S_14_C_24tr_
					925(O_2_^i^)		3:2:2+S_14_C_24tr_
					925×2(O_2_^i^)		3:2:2+S_14_C_24tr_
					950(O_2_^i^)	part.melt	Rav+8:4:5+SC
#3L^g^					900×2		2:2:l+3:2:2+SC
					900×3		2:2:1+3:2:2+SC
	42.5	47.5	10	800			S_2_B_2_+Rav
					875		2:2:1+S_2_B_2_+Tet
					925	comp.melt	Rav+Tet
	42.16	32.35	25.49	700			
				750			
				850			
					875		2:2:1+3:2:2+ S_14_C_24_+SC
					900		2:2:1+3:2:2+S_14_C_24tr_+SC_tr_
	42	40	18	700			
				850			
					875		2:2:1 + S_3_B_2_+S_14_C_24tr_

a Starting materials: SrC0_3_, Bi_2_0_3_,CuO, except when listed in italics. Compositions given in italics were formulated from the listed prereacted compounds or compositions. S.B.=Sr_1.2407_Bi_1.2222_O_3.074_, Rhomb=SrBi_2.75_O_5.125_, Tet=SrBi_1.22_O_2.83_.

b Specimens were given all previous heat treatments listed in the initial column, sequentially, and held at temperature 16–24 h, with grinding in-between, for the number of times shown and then reheated at the final temperature overnight. Specimens were heated as pellets on Au foil or MgO single crystal plates, except as indicated. In general, only a small portion of the specimen used for the initial (calcined) heat treatments was used to make sequential “final” heat treatments. Q=quenched.

c Compounds are listed in order of estimated amounts, most prevalent first.
tr=trace, just barely discernible B_2_C=Bi_2_CuO_4_ S_2_C=Sr_2_CuO_3_ SC=SrCuO_2_ S_l4_C_24_ = Sr_14_CU_24_O_41_ Rhomb=rhombohedral solid solution SB_2_=SrBi_2_O_4_ Tet=Tetragonal solid solution near SrBi_1.22_O_2.83_ S_2_B_2_=Sr_2_Bi_2_O_5_ S_3_B_2_ = Sr_3_Bi_2_O_6_ S_3_B = Sr_6_Bi_2_O_9_ 2:2:l = Sr_2_Bi_2_CuO_6_ Rav=Raveau-type solid solution, ~Sr_1.8−_*_x_*Bi_2.2+_*_x_*CuO_2_ 8:4:5=Sr_8_Bi_4_Cu_5_O_19+_*_x_* 3:2:2 = Sr_3_Bi_2_Cu_2_O_8_ X,unk=phases of unknown composition “7:2:2”=unknown phase, probably oxycarbonate with diffraction peaks a ~ 18.40 ° and —21.27 ° 2*θ* *=unknown phase, probably an oxycarbonate, with diffraction peaks a 4.40 ° and 5.68 ° plus major peaks at 30.50 ° and 32.45 ° 2*θ*

d These specimens are numbered when more than one batch of a given oxide ratio were prepared.

e Specimens were heated in 70Ag/30Pd tubes, which caused the appearance of unknown phases due to reaction with the tube.

f Specimens were contained in 3-mm diameter Au tubes. Excessive heat treatment in such tubes resulted in appreciable loss of Cu to the surrounding Au tube.

g L=Specimen prepared by an organic precursor route utilizing lactic acid.

h The specimen was melted in an A1_2_0_3_ crucible and poured onto an A1 chill plate.

i Specimen heated in one atmosphere pure oxygen instead of in air.

j Increase in amount of S_14_C_24_ relative to 3:2:2; indicates that the 3:2:2 phase is not favored by higher oxygen partial pressure.

k Specimen cooled from 925 to 889 °C at 1 °C/h.

l Amount of 2:2:1 phase not increased.

m Specimen heated in atmosphere of mixed Argon/Oxygen with the partial pressure of oxygen equal to 0.15 atm; amount of 2:2:1 phase greatly increased.

n Amount of 2:2:1 phase increased relative to previous heat treatment.

p This specimen was prepared as described in reference [30].

**Table 1b:** Experimental conditions for crystal growth experiments

Charge	Flux	Container	Temperature cycle	Results
SrO:1/2Bi_2_O_3_ 4:1	(KNa)C1	sealed small diameter Au	800 °C 16 h	
98 wt%	2 wt%			
SrO:1/2Bi_2_O_3_ 4:1	(KNa)C1	sealed small diameter Au	800 °C 16 h	
90 wt%	10 wt%			
SrO:1/2Bi_2_O_3_ 4:1	(KNa)C1	sealed small diameter Au	1025→650 °C @5°C/h	
80 wt%	20 wt%			
Sr_6_Bi_2_O_9_		open small diameter Au	925→900 °C @ 0.3 °C/h	
Sr_6_Bi_2_O_9_	(KNa)C1	sealed small diameter Au	900 °C 16 h	
98 wt%	2 wt%			
Sr_6_Bi_2_O_9_	(KNa)C1	sealed small diameter Au	800 °C 16 h	
98 wt%	2 wt%			
Sr_6_Bi_2_O_9_	(KNa)C1	sealed small diameter Au	800 °C 16 h	S_3_B oxychloride
90 wt%	10 wt%			
Sr_6_Bi_2_O_9_	(KNa)C1	sealed small diameter Au	1025→650 °C @ 5 °C/hr	S_3_B_2_ xtls hydrate after long exposure to air
80 wt%	20 wt%			
Sr_6_Bi_2_O_9_ 80 wt%	(KNa)C1 20 wt%	sealed small diameter Au	950→650 °C @4°C/h	
SrO:1/2 Bi_2_O_3_ 2:1	(KNa)C1	sealed small diameter Au	800 °C 16 h	
98 wt%	2 wt%			
SrO:1/2Bi_2_O_3_ 2:1	10 wt%	sealed small diameter Au	800 °C 16 h	
90 wt%	10 wt%			
Sr_2_Bi_2_O_5_		sealed small diameter Ft	925 °C 162 h	S_2_B_2_ Partially melted
Sr_2_Bi_2_O_5_		sealed small diameter Au	1025→950 °C @ 1°A	b.c. Tet
Sr_2_Bi_2_O_5_		sealed small diameter Au	1025→900 °C @1°C/h	b.c. Tet
Sr_2_Bi_2_O_5_		sealed small diameter Au	1025→900 °C @ 1 °/h; 875 °C−225 h	S_2_B_2_
Sr_2_Bi_2_O_5_ 98 wt%	(KNa)C1 2wt%	sealed small diameter Au	900→640°C @3°C/h	S_2_B_2_
Sr_2_Bi_2_O_5_ 90wt%	(KNa)C1 10wt%	sealed small diameter Au	900→640 °C @3°C/h	S_2_B_2_
Sr_2_Bi_2_O_5_ 80wt%	(KNa)C1 20wt%	sealed small diameter Au	900→640 °C @3°C/h	
Sr_2_Bi_2_O_5_ 50 wt%	(KNa)C1 50wt%	sealed small diameter Au	900→640 °C @3°C/h	
Sr_2_Bi_2_O_4_ 80wt%	(KNa)C1 20wt%	sealed large diameter Au	900→850 °C @3°C/h	
Sr_2_Bi_2_O_4_ 80wt%	(KNa)C1 20wt%	sealed large diameter Au	900→700 °C @3°C/h	
Sr_2_Bi_2_O_4_ 20wt%	(KNa)C1 80wt%	sealed small diameter Au	800→645 °C @ 1*°*C/h	SB_2_
Sr_2_Bi_2_O_4_ 50 wt%	(KNa)C1 50wt%	sealed small diameter Au	800→645 °C @ 1°C/h	SB_2_
Sr_2_Bi_2_O_4_ 20wt%	(KNa)C1 80wt%	sealed Pt	740→570 °C @6°C/h	SB_2_
SrO:1/2Bi_2_O_3_:CuO 3:1:1 90 wt%	(KNa)C1 10wt%	sealed small diameter Au	900 °C 16 h	xtals soluble inH_2_O
SrO:1/2Bi_2_O_3_:CuO 2:1:1		large diameter Pt	950→615 °C @ 1 °C/min	
SrO:1/2Bi_2_O_3_:CuO 2:1:1 90wt%	(KNa)C1 10wt%	sealed small diameter Au	900 °C 16 h	
SrO:1/2Bi_2_O_3_:CuO 2:1:1 90wt%	(KNa)C1 10wt%	sealed small diameter Au	900→650 *°*C *@*3°C/h	partially melted needlelike xtals of 8:4:5
SrO:1/2Bi_2_O_3_:CuO 2:1:1 90 wt%	2NaF:SrF_2_ 50.86:49.14 10wt%	sealed small diameter Au	900→650 °C 3°C/h	Partially melted Rav
SrO:1/2Bi_2_O_3_:CuO 45 :45 : 10		Ag/Pd small diameter tube	950→800 °C @ 1 °C/h	
Sr_3_Bi_2_Cu_2_O_8_ 90 wt%	(KNa)C1 10wt%	sealed small diameter Au	900 °C 16 h	xtals not soluble in H_2_O
SrO:1/2Bi_2_O_3_:CuO 42.5 : 47.5 : 10		Ag/Pd small diameter tube	950→800 °C @1°C/h	
SrO:1/2Bi_2_O_3_:CuO 41:41:18		sealed small diameter Au	925→900 °C @ 1°C/h	
SrO:1/2Bi_2_O_3_:CuO 41 : 40 : 19		open small diameter Au	900→450 °C @ 1°C/h	
SrO:1/2Bi_2_O_3_:CuO 40.5:49.5:10		Ag/Pd small diameter tube	950→800 °C @1°C/h	
SrO:1/2Bi_2_O_3_:CuO 40.5:40.5:19		sealed small diameter Au	925→900 °C @ 1°C/h	2:2:1 + Rav
Sr_2_Bi_2_CuO_6_		Ag/Pd small diameter tube	950→800 °C @1°C/h	Rav+Tet
Sr_2_Bi_2_CuO_6_		Ft small diameter tube	950→800 °C @1°C/h	
Sr_2_Bi_2_CuO_6_		sealed small diameter Au	950→800 °C @ 1°C/h	Rav
Sr_2_Bi_2_CuO_6_		open small diameter Au	950→400 °C @ 1°C/h	
Sr_2_Bi_2_CuO_6_ 90 wt%	(KNa)C1 10 wt%	sealed small diameter Au	900 °C 16 h	Rav completely melted
Sr_2_Bi_2_CuO_6_ 98wt%	NaF:KF 42:58 2wt%	sealed small diameter Au	900 °C 3 d	Rav
Sr_2_Bi_2_CuO_6_ 90 wt%	NaF:KF 42:58 10 wt %	sealed small diameter Au	900→650 °C @3°C/h	Rav
Sr_2_Bi_2_CuO_6_ 90 wt%	2NaF:SrF_2_ 50.86:49.14 10wt%	sealed small diameter Au	850→650 °C @3°C/h	Rav
SraBi_2_CuO_6_ 90wt%	2NaF:CaF_2_ 51.73:48.28 10 wt%	sealed small diameter Au	900→650 °C 3°C/h	Rav
SrO:1/2Bi_2_O_3_:CuO 3:2:3 80 wt%	(KNa)C1 20 wt%	sealed small diameter Au	1025→650 °C @ 5 °C/min	
SrO:1/2Bi_2_O_3_:CuO 36 :44 :20 Rav		Ag/Pd small diameter tube	950→800°C @ 1°C/h	
SrO:1/2Bi_2_O_3_:CuO 1:1:1		large diameter Ft	950→615°C @ 1 °C/min	
SrO:1/2Bi_2_O_3_:CuO 1:1:1 80wt%	(KNa)C1 20 wt%	sealed small diameter Au	1025→650 °C @ 5 °C/min	

**Table 2:** Crystallographic data

Phase formula	Unit cell parameters (Å)				Symmetry	Space group	Reference
	*a*	*b*	*c*	*β*°			
Bi_2_CuO_4_	8.510		5.814		Tet	P4/ncc	23
SrCuO_2_	3.5730(2)	16.3313(8)	3.9136(2)		Orth	Cmcm	26
Sr_2_CuO_3_	3.4957	12.684	3.9064		Orth	Immm	JCPDS^a^ 34–283
Sr_14_CU_24_O_41_	11.483(1)	13.399(1)	3.9356(3)^b^		Orth	Fmmm	This work
~Rhomb-SS^c^	3.979		28.51		Rhomb		27d
Sr*_x_*Bi_1−_*_x_*,O_(3−x)/2_							
0.1⩽ *x* ⩽0.265							
SrBi_2_O_4_	19.301(2)	4.3563(5)	6.1049(7)	94.85(1)	Men	C2/m	This work
~Tet-SS SrBi_1.22_O_2.83_	13.239(2)		4.257(1)		Tet	I4/m	27
Sr_2_Bi_2_O_5_	3.8262(2)	14.307(1)	6.1713(4)		Orth	Cmcm	This work
Sr_3_Bi_2_O_6_	12.526(1)		18.331(2)		Rhomb	$R\bar{3}m$	This work
Sr_3_Bi_2_O_9_	6.009		58.663		Rhomb^c^		This work
Sr_2_Bi_2_CuO_6_	24.493(2)	5.4223(5)	21.959(2)	105.40(1)	Mon	C2/m	18
Raveau-SS Sr_1.8−_*_x_*Bi_2.2+_*_x_*Cu_1±_*_x_*_/2_O*_z_*	26.889(9)	5.384(2)	26.933(8)	113.67(3)	Mon	C2	This work^f^
0⩽*x* ⩽0.15							
Sr_8_Bi_4_Cu_5_O_19+_*_x_*	33.991(3)	24.095(2)	5.3677(5)		Orth	Fmmm	This work
Sr_3_Bi_2_Cu_2_O_8_	24.937(7)	5.395(2)	19.094(7)	96.97(3)	Mon	C2/m	This work

a Joint Committee for Powder Diffraction Standards, X-Ray Diffraction card file.

b Contains superstructure with c′=7c.

c -SS=solid solution.

d Unit cell dimensions for x=0.19.

e Apparently a subcell.

f Unit cell dimensions for x=0.

**Table 3:** X-ray powder diffraction data for Sr_14_Cu_24_O_41_

*d* obs(Å)	Rel *I*(%)	2*θ* obs	2*θ* calc^a^	*hkl*
6.68	2	13.25	13.22	020
5.72	<1	15.48	15.45	200
4.352	2	20.39	20.38	220
3.596	6	24.74	24.75	111
3.347	12	26.61	26.61	040
3.021^b^	1	29.55		
2.8879	100	30.94	30.91	240
2.8608	66	31.24	31.22	131
2.6853	52	33.34	33.30	311
2.6339	10	34.01	34.00	420
2.6049^b^	1	34.40		
2.4245^b^	1	37.05		
2.3364	38	38.50	38.47	331
2.2834^b^	1	39.43		
2.2324	1	40.37	40.39	060
2.1742	42	41.50	41.47	151
2.0801	1	43.47	43.48	260
1.9878^b^	3	45.60		
1.9718	13	45.99	45.96	002
1.9582	6	46.33		
1.9103	14	47.56	47.55	600
1.8920	6	48.05	48.04	022
1.8657	2	48.77	48.78	202
1.8361	17	49.61	49.57	620
1.8108	46	50.35	50.32	531
1.7975	3	50.75	50.76	222
1.7610	2	51.88	51.87	460
1.7413^b^	2	52.51		
1.7096^b^	2	53.56		
1.7026	3	53.80	53.81	171
1.6733	15	54.82	54.81	080
1.6599	2	55.30	55.32	640
1.6290	16	56.44	56.42	642
1.5934	9	57.82	57.82	551
1.5789	2	58.40	58.39	422
1.5696	13	58.78	58.79	371
1.5542^b^	1	59.42		
1.5117^b^	1	61.27		
1.5037	1	61.63	61.65	711
1.4783	4	62.81	62.82	062
1.4624	11	63.57	63.59	442
1.4518	9	64.09	64.11	660
1.4422^b^	3	64.57		
1.4327	15	65.05	65.05	731
1.4017	5	66.67	66.69	820
1.3731	11	68.25	68.28	602
1.3450	4	69.88	69.90	622

a Calculated from an orthorhombic unit cell, *a* = : 11.466(2); *b* = 13.389(2) and *c* = 3.9458(6) Å.

b Superstructure peak.

**Table 4:** X-ray powder diffraction data for the compound SrBi_2_O_4_

*d* obs(Å)	Rel *I*(%)	2*θ* obs	2*θ* calc^a^	*hkl*
9.64	9	9.17	9.19	200
6.09	4	14.53	14.55	001
5.36	1	16.54	16.56	$20\bar{1}$
4.813	22	18.42	18.44	400
3.626	7	24.53	24.53	401
3.606	6	24.67	24.69	310
3.454	29	25.77	25.78	111
3.205	97	27.81	27.81	600
3.168	100	28.15	28.16	$31\bar{1}$
3.040	93	29.36	29.38	311
2.9743	15	30.02	30.03	$20\bar{2}$
2.9417	3	30.36	30.38	$60\bar{1}$
2.8326	6	31,56	31.57	202
2.7421	13	32.63	32.63	601
2.6728	7	33.50	33.51	$51\bar{1}$
2.5454	1	35.23	35.25	511
2.4781	7	36.22	36.23	402
2.4526	3	36.61	36.63	112
2.4051	1	37.36	37.38	800
2.3065	19	39.02	39.03	$60\bar{2}$
2.2724	5	39.63	39.64	312
2.1782	34	41.42	41.42	020
2.1196	22	42.62	$\{\begin{array}{c} 42.59\\ 42.64 \end{array}$	711 602
2.0501	1	44.14	44.13	021
2.0291	2	44.62	44.62	512
2.0197	3	44.84	44.86	$20\bar{3}$
1.9841	5	45.69	45.69	420
1.9686	5	46.07	46.07	$80\bar{2}$
1.9191	19	47.33	47.35	910
1.8701	33	48.65	48.63	$91\bar{1}$
1.8427	8	49.42	49.40	$11\bar{3}$
1.8145	17	50.24	50.25	403
1.8018	43	50.62	50.63	620
1.7909	30	50.95	50.95	911
1.7705	16	51.58	51.57	022
1.7569	11	52.01	52.00	$22\bar{2}$
1.7318	9	52.82	52.81	313
1.7270	8	52.98	53.00	222
1.7096	10	53.56	53.53	$51\bar{3}$
1.7058	12	53.69	53.70	621
1.6812	3	54.54	54.54	$91\bar{2}$
1.6514	2	55.61	55.60	603
1.6357	5	56.19	56.18	422
1.6107	6	57.14	57.12	513
1.6023	11	57.47	57.45	12,0,0
1.5831	19	58.23	58.22	622
1.5691	7	58.80	58.77	912
1.5670	6	58.89	58.90	10,0,2

a Calculated on the basis of a monoclinic cell, C2/m, *a* = 19.301(2), *b*=4.3563(5), *c* = 6.1049(7) Å, *β*=94.85(1)°.

**Table 5:** X-ray powder diffraction data for the compound Sr_2_Bi_2_O_5_

*d* obs(Å)	Rel *I*(%)	2*θ* obs	2*θ* calc^a^	*hkl*
7.161	17	12.35	12.3	020
4.676	15	18.96	18.98	021
3.697	32	24.05	24.06	110
3.171	1	28.11	28.12	111
3.094	100	28.84	28.83	041
2.9842	10	29.92	29.92	130
2.8319	8	31.57	31.55	022
2.6865	23	33.33	33.32	131
2.3857	1	37.67	37.69	060
2.3684	11	37.96	37.95	112
2.3373	<1	38.49	38.50	042
2.2918	9	39.28	39.29	150
2.2254	2	40.50	40.52	061
2.1466	26	42.06	42.09	132
1.9767	1	45.88	45.87	023
1.9122	8	47.51	47.49	200
1.8873	2	48.18	48.19	062
1.8401	8	49.50	49.51	152
1.8030	5	50.59	50.59	170
1.7979	8	50.74	50.75	113
1.7827	17	51.20	51.19	043
1.7712	2	51.56	51.58	221
1.7306	7	52.86	52.86	171
1.6936	5	54.11	54.10	133
1.6873	1	54.33	54.34	240
1.6271	17	56.51	56.51	241
1.5849	<1	56.16	58.14	222
1.5570	5	59.31	59.32	172
1.5472	7	59.72	59.71	082
1.5424	7	59.92	59.91	004

a Calculated on the basis of an orthorhombic unit cell, Cmcm, *a* = 3.8262(2), *b* = 14.307(1), *c* =6.1713(4) Å.

**Table 6:** X-ray powder diffraction data for the compound Sr_3_Bi_2_O_6_

*d* obs(Å)	Rel *I*(%)	2*θ* obs	2*θ* calc^a^	*hkl*
9.32	2	9.48	9.47	101
6.997	2	12.64	12.63	012
6.100	4	14.51	14.49	003
4.662	16	19.02	19.00	202
4.371	14	20.30	20.29	113
4.217	8	21.05	21.03	104
4.001	11	22.20	22.20	211
3.740	9	23.77	23.76	122
3.1326	100	28.47	28.48	220
3.0394	85	29.36	29.38	205
2.9694	6	30.07	30.08	131
2.8582	4	31.27	31.27	312
2.7861	3	32.10	32.09	223
2.7454	2	32.59	32.58	116
2.7347	2	32.72	32.74	125
2.6013	7	34.45	34.46	042
2.5150	8	35.67	35.67	134
2.4024	1	37.40	37.42	232
2.3588	26	38.12	38.13	027
2.3329	2	38.56	38.54	404
2.3265	2	38.67	38.68	315
2.2420	3	40.19	40.19	018
2.2073	5	40.85	$\{\begin{array}{c} 40.85\\ 40.86 \end{array}$	413 217
2.1797	63	41.39	41.38	045
2.1552	2	41.88	41.90	051
2.1111	2	42.80	$\{\begin{array}{c} 42.79\\ 42.81 \end{array}$	502 208
2.0377	2	44.42	$\{\begin{array}{c} 44.43\\ 44.44 \end{array}$	241 009
2.0011	11	45.28	$\{\begin{array}{c} 45.29\\ 45.30 \end{array}$	422 128
1.9767	6	45.87	$\{\begin{array}{c} 45.90\\ 45.91 \end{array}$	333 137
1.9376	7	46.85	$\{\begin{array}{c} 46.86\\ 46.87 \end{array}$	511 119
1.9062	4	47.67	47.68	152
1.8832	12	48.29	48.27	407
1.8711	7	48.62	$\{\begin{array}{c} 48.62\\ 48.62 \end{array}$	244 416
1.8230	9	49.99	49.99	318
1.8087	24	50.41	50.44	600
1.7893	46	51.00	51.00	425
1.7753	9	51.43	$\{\begin{array}{c} 51.44\\ 51.45 \end{array}$	431 309
1.7512	3	52.19	$\{\begin{array}{c} 52.21\\ 52.22 \end{array}$	342 048
1.7367	40	52.66	52.66	0,2,10
1.7248	4	53.05	53.09	336
1.7200	4	53.21	53.20	155
1.6855	5	54.39	54.38	238
1.6146	9	56.99	57.00	247
1.5931	4	57.83	57.84	2,0,11
1.5667	24	58.90	58.94	440
1.5569	5	59.31	$\{\begin{array}{c} 59.35\\ 59.35 \end{array}$	164 606
1.5443	9	59.84	$\{\begin{array}{c} 59.85\\ 59.86 \end{array}$	701 419

a Calculated on the basis of a rhombohedral unit cell *a* = 12.526(1), *c* = 18.331(2) Å.

**Table 7:** X-ray powder diffraction data for the compound Sr_6_Bi_2_O_9_

*d* obs(Å)	Rel *I*(%)	2*θ* obs	2*θ* calc^a^	*hkl*^b^
4.891	18	18.12	18.13	0,0,12
4.777	1	18.56		
4.397	1	20.18		
4.258	12	20.85	20.93	018
4.197	6	21.15		
3.810	1	23.33		
3.589	1	24.79		
3.396	3	26.22	26.13	1,0,13
3.318	1	26.85		
3.271	1	27.24		
3.218	1	27.70		
3.184	1	28.00		
3.092	1	28.85		
3.0105	58	29.65	29.74	110
2.9997	61	29.76	29.79	1,0,16
2.9859	100	29.90		
2.8779	1	31.05		
2.8493	1	31.37		
2.7283	1	32.80		
2.6437	5	33.88	33.74	1,0,19
2.5615	16	35.00	35.05	1,1,12
2.5357	9	35.37		
2.4827	2	36.15		
2.4436	4	36.75	36.74	0,0,24
2.4075	1	37.32		
2.3829	2	37.72		
2.3672	2	37.98		
2.3383	1	38.55		
2.2974	1	39.18		
2.2603	6	39.85	39.99	0,2,13
2.2308	1	40.40		
2.1272	32	42.46	42.60	0,2,16
2.0953	15	43.14		
2.0452	2	44.25		
2.0146	1	44.96		
1.9952	4	45.42		
1.9845	3	45.68		
1.9550	4	46.41		
1.9502	6	46.53		
1.9415	8	46.75		
1.9337	10	46.95		
1.9054	4	47.69		
1.9006	5	47.82		
1.8629	4	48.85		
1.8452	2	49.35		
1.8118	2	50.32		
1.8001	3	50.67		
1.7509	3	52.20		
1.7364	18	52.67		
1.7318	35	52.82	52.77	300
1.7188	21	53.25		
1.7031	2	53.78		
1.6838	2	54.45		
1.6557	2	55.45		
1.6354	9	56.20		
1.6295	4	56.42		
1.6156	3	56.95		
1.5884	1	58.02		
1.5802	2	58.35		
1.5600	2	59.18		

a Calculated on the basis of a rhombohedral subcell with *a* = 6.009, *c* = 58.663 Å.

b Based on the intensities observed in single crystal precession photographs, figure 11.

**Table 8:** X-ray powder diffraction data for the Raveau-type phase at the composition Sr_1.8_Bi_2.2_CuO_6.1_^a^

*d* obs(Å)	Rel *I*(%)	2*θ* obs	2*θ* calc^b^	*hkl*^c^
12.35	6	7.15	7.17	200
6.16	1	14.37	14.38	400
5.47	1	16.20	16.17	401
5.26	3	16.83	16.84	110
4.50	1	19.70	19.70	310
4.348	2	20.41	20.44	$60\bar{1}$
4.183	2	21.22	21.22	$11\bar{4}$
4.105	34	21.63	21.63	600
3.761	2	23.64	$\{\begin{array}{c} 23.62\\ 23.62 \end{array}$	$\begin{array}{c} 31\bar{5}\\ 11\bar{5} \end{array}$
3.632	4	24.49	$\{\begin{array}{c} 24.45\\ 24.47 \end{array}$	$\begin{array}{c} 51\bar{4}\\ 510 \end{array}$
3.457	58	25.75	$\{\begin{array}{c} 25.76\\ 25.78 \end{array}$	$\begin{array}{c} 51\bar{5}\\ 115 \end{array}$
3.384	1	26.32	26.32	$11\bar{6}$
3.239	4	27.52	27.50	$51\bar{6}$
3.220	6	27.68	27.70	$80\bar{1}$
3.092	24	28.85	28.85	$71\bar{4}$
3.081	66	28.96	28.96	800
3.013	100	29.63	$\{\begin{array}{c} 29.62\\ 29.64 \end{array}$	$\begin{array}{c} 71\bar{5}\\ 315 \end{array}$
2.9427	5	30.35	30.32	710
2.9380	5	30.40	30.41	$20\bar{9}$
2.9025	11	30.78	30.81	$71\bar{6}$
2.7929	3	32.02	32.05	514
2.7462	2	32.58	32.54	316
2.6924	58	33.25	$\{\begin{array}{c} 33.24\\ 33.25 \end{array}$	$\begin{array}{c} 4,0,\bar{10}\\ 020 \end{array}$
2.6317	2	34.04	$\{\begin{array}{c} 34.04\\ 34.05\\ 34.06 \end{array}$	$\begin{array}{c} 6,0,\bar{10}\\ 2,0,\bar{10}\\ 220 \end{array}$
2.5831	7	34.70	$\{\begin{array}{c} 34.68\\ 34.70 \end{array}$	$\begin{array}{c} 91\bar{5}\\ 515 \end{array}$
2.5560	2	35.08	35.11	$10,0,\bar{1}$
2.4623	15	36.46	36.45	10,0,0
2.4481	5	36.68	36.71	$4,0,\bar{11}$
2.4182	5	37.15	37.15	$6,0,\bar{11}$
2.3565	5	38.16	38.12	10,0,1

a Oxygen content not certain.

b Calculated from monoclinic unit cell *a*=26.889(9), *b*=5.384(2), *c* =26.933(3) Å, *β*= 113.67(3)°.

c Indexed based on single crystal *F*_obs_ data received from M. Onoda [34].

**Table 9:** X-ray powder diffraction data for the compound Sr_8_Bi_4_Cu_5_O_19+_*_x_*^a^

*d* obs(Å)	Rel *I*(%)	2*θ* obs	2*θ* calc^b^	*hkl*^c^
17.05	3	5.18	5.20	200
12.08	3	7.31	7.33	020
9.85	1	8.97	8.99	220
5.668	<1	15.62	15.63	600
4.425	2	20.05	20.05	131
4.253	2	20.87	20.89	800
4.153	2	21.38	21.39	511
4.015	4	22.12	22.12	060
3.911	3	22.72	22.73	260
3.729	13	23.84	23.83	531
3.559	1	25.00	24.98	711
3.418	2	26.05	26.04	351
3.288	33	27.10	27.12	731
3.173	23	28.10	28.11	551
3.011	27	29.65	29.64	080
2.9665	4	30.10	30.11	280
2.8861	100	30.96	30.95	171
2.8317	11	31.57	31.56	12,0,0
2.7569	2	32.45	32.44	12,2,0
2.6837	35	33.36	33.36	002
2.6498	1	33.80	33.78	202
2.6182	3	34.22	34.20	022
	4	34.40^d^		
2.5903	1	34.60	34.62	222
2.4881	2	36.07	36.07	771
2.4264	27	37.02	37.00	14,0,0
2.4080	24	37.31	37.29	0,10,0
2.3793	3	37.78	37.77	14,2,0
2.3417	1	38.41	38.42	11,5,1
2.3145	1	38.88	38.88	12,6,0
2.2571	2	39.91	39.93	13,3,1
2.2303	1	40.41	40.39	062
2.2125	1	40.75	40.75	262
2.1485	2	42.02	42.02	791
2.1244	4	42.52	42.52	16,0,0
2.1135	3	42.75	42.77	13,5,1
2.0639	14	43.83	43.84	12,8,0
2.0077	14	45.12	45.12	0,12,0
2.0036	14	45.22	45.22	082
1.9474	24	46.60	46.58	12,0,2
1.9443	23	46.68	46.70	5,11,1
1.9164	20	47.40	47.42	15,5,1
1.8909	2	48.08	48.10	14,8,0
1.8715	21	48.61	48.61	7,11,1
1.8351	8	49.64	49.63	12,10,0
1.8001	19	50.67	50.66	14,0,2
1.7935	21	50.87	50.89	0,10,2
1.7875	12	51.05	51.08	9,11,1
1.7525	3	52.15	52.14	12,6,2
1.7494	4	52.25	52.24	1,13,1
1.7264	3	53.00	53.02	513
1.7099	9	53.55	53.54	14,10,0
1.6967	7	54.00	54.01	5,13,1
1.6915	7	54.18	54.18	533
1.6604	10	55.28	55.28	19,3,1
1.6478	27	55.74	55.73	7,13,1
1.6437	21	55.89	55.90	733
1.6359	10	56.18	56.18	12,8,2
1.6288	5	56.45	56.46	553
1.6007	25	57.53	57.53	19,5,1
1.5851	26	58.15	58.14	753
1.5453	13	59.80	$\{\begin{array}{c} 59.79\\ 59.81 \end{array}$	14,8,2 22,0,0

a Oxygen content based on structure derived by [40].

b Calculated by least-square analysis from orthorhombic unit cell, Fmmm, *a* =33.991(3), *b*=24.095(2), *c* = 5.3677(5) Å.

c Indexed with the aid of the single crystal precession photographs, figure 17 and intensities calculated from the published structure [40].

d SrCuO_2_.

**Table 10:** X-ray powder diffraction data for the compound Sr_3_Bi_2_Cu_2_O_8_^a^

*d* obs(Å)	Rel *I*(%)	2*θ* obs	2*θ* calc^b^	*hkl*
24.7^c^	1	3.57		
12.35	3	7.15	7.14	200
5.26	2	16.84	16.81	110
5.12	1	17.32	17.33	$11\bar{1}$
4.120	10	21.55	21.52	600
4.064^c^	2	21.85		
3.992	2	22.25	22.22	113
3.625	9	25.54	24.54	602
3,573	2	24.90	24.92	$11\bar{4}$
3.315	48	26.87	26.86	$60\bar{4}$
3.124	11	28.55	28.63	$11\bar{5}$
3.095	33	28.82	28.83	800
3.053	2	29.20	29.23	$80\bar{2}$
3.043	2	29.33	29.32	513
2.9220	100	30.57	30.57	$80\bar{3}$
2.8031	1	31.90	31.79	315
2.7082	26	33.05	33.06	007
2.6963	60	33.20	33.19	020
2.6324	4	34.03	34.04	$71\bar{4}$
2.5581	2	35.05	35.07	$22\bar{2}$
2.5518	2	35.14	35.11	$80\bar{5}$
2.5281	3	35.48	35.56	222
2.4748	20	36.27	36.26	10,0,0
2.4384	16	36.83	36.85	$91\bar{2}$
2.3933	3	37.55	37.55	$31\bar{7}$
2.2571	3	39.91	39.91	317
2.0993	2	43.05		
2.0629	5	43.85		
2.0334	34	44.52		
1.9877	4	45.60		
1.9815	3	45.75		
1.9125	41	47.50		
1.8919	2	48.05		
1.8539	2	49.10		
1.8239	13	49.96		
1.8090	14	50.40		
1.7908	3	50.95		
1.7875	2	51.05		
1.7360	5	52.68		
1.7232	3	53.10		
1.6857	18	54.38		
1.6532	12	55.54		
1.6388	4	56.07		
1.6279	18	56.48		
1.5971	24	57.67		
1.5744	19	58.58		
1.5620	8	59.09		
1.5475	6	59.70		

a Heated to 925 °C in flowing O_2_ on Au foil. Total oxygen content uncertain.

b Calculated on the basis of a C-centered monoclinic cell with *a* = 24.937(7), *b* = 5.395(2), *c* = 19.094(7) Å, *β* =96.97(3)°

c Superstructure peaks.

## References

[1] Bednorz JG, Müller KA (1986). Z Phys B-Condensed Matter.

[2] Takagi H, Uchida S, Kitazawa K, Tanaka T (1987). Jap J Appl Phys Lett.

[3] Cava RJ, VanDover RB, Batlog B, Rietman EA (1987). Phys Rev Lett.

[4] Wu MK, Asburn JR, Torng CJ, Hor PH, Meng RL, Gao L, Huang ZJ, Wang YQ, Chu CW (1987). Phys Rev Lett.

[5] Cava RJ, Batlog B, VanDover RB, Murphy DW, Sunshine SA, Siegrist T, Remeika JR, Rietman EA, Zahurak S, Espinosa GP (1987). Phys Rev Lett.

[6] Roth RS, Davis KL, Dennis JR (1987). Ad Ceram Mat.

[7] Roth RS, Rawn CJ, Beech F, Whitler JD, Anderson JO, Yan MF (1988). Ceramic Superconductors II. Amer Ceram Soc.

[8] Maeda H, Tanaka Y, Fukutomi M, Asano T (1988). Jap J Appl Phys.

[9] Sheng ZZ, Hermann AM (1988). Nature.

[10] Subramanian MA, Torardi CC, Gopalakrishnan J, Gai PL, Calabrese TC, Askew TR, Flippen RB, Sleight AW (1988). Science.

[11] Cava RJ, Batlogg B, Krajewski JJ, Rupp LW, Schneemeyer LF, Siegist T, vanDover RB, Marsh P, Peck WF, Gallegher PK, Glarum SH, Marshall JH, Farrow RC, Waszczak JV, Hull R, Treor P (1988). Nature.

[12] Cava RJ, Batlogg B, Kajewski JJ, Farrow R, Rupp LW, White AE, Short K, Peck WF, Kometari T (1988). Nature.

[13] Tokura Y, Takagi H, Uchida S (1989). Nature.

[14] Roth RS, Rawn CJ, Ritter JJ, Burton BP (1989). J Amer Ceram Soc.

[15] Siegrist T, Zahurak SM, Murphy DW, Roth RS (1988). Nature.

[16] Roth RS, Rawn CJ, Whitler JD, Chiang CK, Wong-Ng WK (1989). J Amer Ceram Soc.

[17] Siegrist T, Schneemeyer LF, Sunshine SA, Waszczak JV, Roth RS (1988). Mat Res Bull.

[18] Roth RS, Rawn CJ, Bendersky LA (1990). J Mater Res.

[b19-jresv95n3p291_a1b] 19Roth, R. S., Rawn, C. J., Burton, B. P., and Beech, F. (to be published).

[b20-jresv95n3p291_a1b] 20Roth, R. S. and Rawn, C. J. (to be published).

[21] Kakhan BG, Lazarev VB, Shaplygin IS (1979). Zh Neorg Khim.

[22] Roth RS, Dennis JR, McMurdie HF (1987). Phase Diagrams for Ceramists. Amer Ceram Soc.

[23] Bovin JC, Thomas D, Tridot G (1973). Compt Rend.

[24] Teske CL, Müller-Bushbaum HZ (1970). Anorg Allg Chem.

[25] Teske CL, Müller-Bushbaum HZ (1969). Anorg Allg Chem.

[26] Wong-Ng WK, McMurdie HF, Paretzkin B, Hubbard CR, Dragoo AL (1988). Powd Diff.

[27] Guillermo R, Conflant P, Bovin JC, Thomas D (1978). Rev Chim Min.

[b28-jresv95n3p291_a1b] 28Huang, N. M., and Roth, R. S. (to be published, J. Amer. Ceram. Soc).

[29] Sillen LG, Aurivillius B (1943). Z Krist.

[30] Levin EM, Roth RS (1964). J Res Natl Bur Stand (US).

[31] Michel C, Hervieu M, Borel MM, Grandin A, Deslandes F, Provost T, Raveau B (1987). Z Phys B.

[32] Torardi CC, Subramanian MA, Calabrese JC, Gopalakrishnan J, McCarron EM, Morriessey KJ, Askew TR, Flippen RB, Chowdhry V, Sleight AW (1988). Phys Rev B.

[33] Torrance JB, Tokura Y, LaPlaca SJ, Huang TC, Savoy RJ, Nazzal AI (1988). Solid State Commun.

[34] Onoda M, Sato M (1988). Solid State Commun.

[b35-jresv95n3p291_a1b] 35Roth, R. S. and Rawn, C. J. (to be published).

[36] Saggio JA, Sugata K, Hahn J, Hwu SJ, Poeppelmeir KR, Mason TO (1989). J Amer Ceram Soc Commun.

[37] Akimitsu J, Yamazaki A, Sawa H, Fujiki H (1987). Jap J Appl Phys.

[38] Chakoumakos BC, Budai JD, Sales BC, Sonder E, Torrance JB, Kitazawa K, Tarascon JM, Jorgensen JR, Thompson M (1989). In High-Temperature Superconductors: Relationships Between Properties, Structure and Solid-State Chemistry. Materials Research Society Symposium Proceedings.

[39] Casais MT, Cascales C, Castro A, de Pedro M, Rasines I, Domarco G, Maza J, Miguelez F, Ponte J, Torron C, Veira JA, Vidal F, Campa JA

[40] Fuertes A, Miravitlles C, Gonzalez-Calbet J, Vallet-Regi M, Obradors X, Rodriguez-Carvajal J (1989). Physica C.

[41] Tarascon JM, LePage Y, Barboux P, Bagley BG, Greene LH, McKinnon WR, Hull GW, Giroud M, Hwang DM (1988). Phys Rev B.

[42] Ikeda Y, Ito H, Shimomura S, Oue Y, Inaba K, Hiroi Z, Takano M (1989). Physica C.

